# Cell Cluster Sorting in Automated Differentiation of Patient-specific Induced Pluripotent Stem Cells Towards Blood Cells

**DOI:** 10.3389/fbioe.2022.755983

**Published:** 2022-05-12

**Authors:** Zhiyao Ma, Marcelo Augusto Szymanskide Toledo, Paul Wanek, Mohamed H. Elsafi Mabrouk, Francis Smet, Rock Pulak, Simon Pieske, Tobias Piotrowski, Werner Herfs, Christian Brecher, Robert H. Schmitt, Wolfgang Wagner, Martin Zenke

**Affiliations:** ^1^ Department of Cell Biology, Institute for Biomedical Engineering, RWTH Aachen University Medical School, Aachen, Germany; ^2^ Helmholtz Institute for Biomedical Engineering, RWTH Aachen University, Aachen, Germany; ^3^ Helmholtz Institute for Biomedical Engineering, Stem Cell Biology and Cellular Engineering, RWTH Aachen University Medical School, Aachen, Germany; ^4^ Union Biometrica, Aalst, Belgium; ^5^ Union Biometrica, Holliston, MA, United States; ^6^ Laboratory for Machine Tools and Production Engineering, RWTH Aachen University, Aachen, Germany; ^7^ Fraunhofer Institute for Production Technology, Aachen, Germany

**Keywords:** cell cluster sorter, large particle flow cytometry, induced pluripotent stem cells, iPS cells, embryoid body, EB formation, hematopoietic cells

## Abstract

Induced pluripotent stem cells (iPS cells) represent a particularly versatile stem cell type for a large array of applications in biology and medicine. Taking full advantage of iPS cell technology requires high throughput and automated iPS cell culture and differentiation. We present an automated platform for efficient and robust iPS cell culture and differentiation into blood cells. We implemented cell cluster sorting for analysis and sorting of iPS cell clusters in order to establish clonal iPS cell lines with high reproducibility and efficacy. Patient-specific iPS cells were induced to differentiate towards hematopoietic cells via embryoid body (EB) formation. EB size impacts on iPS cell differentiation and we applied cell cluster sorting to obtain EB of defined size for efficient blood cell differentiation. In summary, implementing cell cluster sorting into the workflow of iPS cell cloning, growth and differentiation represent a valuable add-on for standard and automated iPS cell handling.

## Graphical Abstract

Clonal iPS cell lines and embryoid bodies (EB) of defined size are obtained by cell cluster sorting in an automated platform.
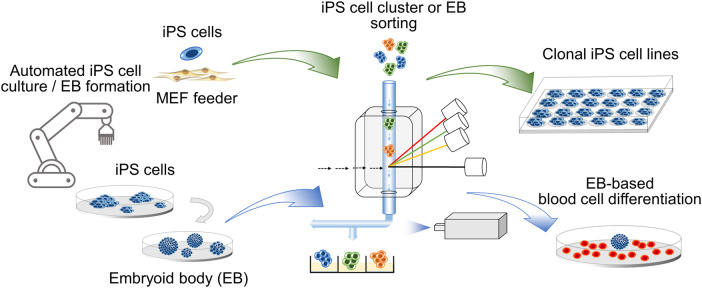

## Introduction

Induced pluripotent stem cells (iPS cells) are engineered stem cells, which are readily obtained from somatic cells of patients by reprogramming (Takahashi and Yamanaka, 2016). They retain the patient-specific genetic background, including disease specific and/or associated mutations. iPS cells offer unprecedented opportunities for disease modeling, drug screening, regenerative and personalized medicine (Rowe and Daley, 2019). However, the processes of iPS cell reprogramming, maintenance, and differentiation are costly and require constant supervision and cell quality assessment by highly trained personnel (Chen et al., 2014; Niessing et al., 2021). Techniques for manual iPS cell handling continue to advance but generating iPS cells of high quality at large scale and in compliance with Good Manufacturing Practice (GMP) still remains a challenge (Baghbaderani et al., 2015; Rivera et al., 2020). Traditional manual handling of iPS cells relies mostly on the expertise of the operator and inevitable inter-technician variabilities affect iPS cell growth and quality. Moreover, manual handling introduces financial and temporal obstacles that limit large scale iPS cell application (Soares et al., 2014; Niessing et al., 2021).

Transferring conventional laboratory processes into industrial manufacturing requires advanced methods and machineries, which are reliable, reproducible, scalable, and adaptable. Automation of distinct steps and integrated systems of iPS cell generation, culture and differentiation have been previously described (Marx et al., 2013; Elanzew et al., 2015; Elanzew et al., 2020; Konagaya et al., 2015; Paull et al., 2015; Coston et al., 2020; Dhingra et al., 2020). For example, an automated culture system maintained iPS cells in their undifferentiated state for up to 60 days (Konagaya et al., 2015). Modular robotic platforms enabled high-throughput reprogramming towards iPS cells and highly parallelized iPS cell cultures and thus, the processing of a large number of iPS cell preparations (Paull et al., 2015; Elanzew et al., 2020). Isolation of individual iPS cell colonies was automated with an integrated working stage composed of robotic picking arm, inverted microscopy and motorized stage (Haupt et al., 2012).

Human iPS cells harbor the potential to develop into all cell types of our body but iPS cell differentiation towards hematopoietic cells has been notoriously challenging (Qin et al., 2014; Ackermann et al., 2015). Frequently, human iPS cells are induced to differentiate into hematopoietic cells through three dimensional embryoid bodies (EB), which recapitulate early steps of human development (Hong et al., 2010; Sturgeon et al., 2014; Garcia-Alegria et al., 2018). Following EB formation specific cocktails of cytokines and/or growth factors in concert with specific stroma cells or scaffolds instruct further development into the desired hematopoietic cell type (Ackermann et al., 2015). EB formation is accomplished 1) by releasing iPS cell clusters from 2D cultures on feeder or feeder-free cultures followed by EB formation in low attachment dishes or 2) by dissociating iPS cell clusters into single cells, which are then reassembled in hanging drops or by centrifugation in spin EB protocols (Ng et al., 2008; Hong et al., 2010; Zeevaert et al., 2020). The EB formation method and EB size impact the differentiated cells obtained (Messana et al., 2008; Hong et al., 2010), which underscores the importance of cell mechanics in EB for differentiation (Zeevaert et al., 2020).

Therefore, in the present work, we embarked on the strategy to automate 1) iPS cell culture on MEF feeder, 2) EB formation from iPS cell clusters and 3) their differentiation into hematopoietic cells. Additionally, we capitalized on cell cluster flow cytometry for 1) analyzing and sorting individual iPS cell clusters to obtain clonal iPS cell lines and 2) sorting EB of defined size for hematopoietic differentiation.

## Materials and Methods

### iPS Cell Lines and iPS Cell Culture

The iPS cell lines used in the present work were derived from an aggressive systemic mastocytosis (ASM) patient by reprogramming peripheral blood CD34^+^ hematopoietic cells using the CytoTune reprogramming kit (Thermo Fisher Scientific) as described in our previous work (patient 1 in Toledo et al., 2021). The iPS cell line 1 (hereafter referred to as line 1) harbors the KIT D816V and NFE2 mutations and displays an erythroid bias upon hematopoietic differentiation. The iPS cell line 2 (hereafter referred to as line 2) does not harbor mutations in the KIT or NFE2 gene and does not show an erythroid bias upon hematopoietic differentiation (Toledo et al., 2021). In the Human Pluripotent Stem Cell Registry (https://hpscreg.eu) line 1 and line 2 are referred to as UKAi004-D and UKAi004-B, respectively. The “unstable” iPS cell line used in the present work was generated as described previously (Toledo et al., 2021) and was classified as “unstable” based on iPS cell morphology and increased number of differentiating cells under standard iPS cell culture condition. iPS cells were cultured on mouse embryo fibroblast (MEF) feeder as described (Sontag et al., 2017; Toledo et al., 2021).

### Establishment of the Liquid Handling Unit (LHU) Modular Preprogrammed Operating System

MEF and iPS cell handling was performed on the automated platform Hamilton STARlet liquid handling unit (LHU) (Hamilton, Reno, NV, United States) with the following functional units: tube carrier, tilt module, plate carriers, tip carriers, shaker, liquid waste container and multiple positions for 6-well plates (Supplementary Figures S1A,B, S2A,B, S3, S4A). Cells were incubated in an automated Cytomat 2 C-LIN incubator (Thermo Fisher Scientific) under normoxic conditions at 37°C and 5% CO_2_. Microscopy was performed with an automated EVOS Fluorescence Digital Inverted Microscope (AMG-Advanced Microscopy Group, WA, United States). Tissue culture plate handling was with a 6-axis robot (VS-087, Denso, Kariya, Japan) mounted on an overhead gantry and encased with a custom-made laminar flow system (Micro CleanRoom Technology, MCRT, Heuchelheim, Germany).

The Hamilton VENUS III software was used to develop the operating system for automated iPS cell culture and differentiation in the LHU. The operating system is composed of several task-specific methods. A number of specific commands were stringed together, forming a method. Methods were edited by the syntax-free graphical method editor. A set of files, including ‘Deck Layout’, ‘Carrier and Labware’, ‘Sequence’, ‘Liquid Class’, and ‘Library’, were linked to the output of the method editor. Every method was associated with several parameters, which can be defined by the user based on the task.

### Evaluation and Optimization of LHU Operating System

MEF feeder layer generation: MEF feeder cells were seeded in 6-well tissue culture plates by LHU (Supplementary Figures S1A,B; Supplementary Figures S4A–C). MEF were then incubated overnight in the automated Cytomat 2 C-LIN incubator and 6-wells were scanned by the automated EVOS Fluorescence Digital Inverted Microscope on the next day. The confluence of MEF feeder was determined by a modified “Phantast” algorithm (Supplementary Figure S4C; see also below). iPS cell growth monitoring: After seeding, several iPS cell colonies were randomly chosen and their increase in size was recorded for eight consecutive days by microscopy (EVOS Fluorescence Digital Inverted Microscope). Collagenase IV treatment: The optimal incubation time for collagenase IV (Gibco) treatment for passaging iPS cells in the LHU was determined by morphology inspection by microscopy. The optimal incubation time was set when most colonies fully detached. Number of resuspension cycles: After collagenase IV treatment, colonies were collected in the LHU and transferred to a 50 ml Falcon tube and broken into smaller cell clusters by pipetting up and down 1 to 8 times (Supplementary Figure S2A). The optimal pipetting cycle number was determined by evaluating cell cluster size. Cell cluster size was calculated by a custom-made Python program “SizeCal” (Supplementary Material S1). Assessment of iPS cell pluripotency: Pluripotency of LHU cultured iPS cells was assessed after 7 days of automatic culture by determining TRA-1-60 expression and comparing to manually cultured iPS cells.

### Generation of iPS Cell Clusters in LHU

Five to 7 days after passaging on MEF feeder layer, iPS cells in 6-well plates were treated in the LHU with dispase (Stem Cell Technologies) for 10–15 min or collagenase IV (Gibco) for 45–60 min. Enzyme was aspirated and 1 ml of IMDM culture medium (Gibco) supplemented with 10% FCS (PAN Biotech) and 100 U/ml penicillin, 100 μg/ml streptomycin (Thermo Fisher Scientific) was added to each well. Colonies were harvested in LHU with custom-made pipetting positions and gently transferred to 50 ml Falcon tubes. The suspension of iPS cell clusters was homogenized by gently pipetting (Supplementary Figure S2A) and submitted to cell cluster sorting with large particle sorter COPAS FP-1000 (Union Biometrica, Holliston, MA, United States).

### TRA-1-60 Live Staining

Live-cell staining of iPS cells in culture with TRA-1-60-Vio488 (Miltenyi Biotec) was performed following manufacturer instructions. Image acquisition was performed with EVOS Fluorescence Digital Inverted Microscope. Alternatively, iPS cells were first treated with collagenase IV for 45 min. iPS cell colonies were gently detached and harvested into a 50 ml Falcon tube, washed twice with KO-DMEM (Thermo Fisher Scientific), stained with TRA-1-60-Vio488 as above and subjected to analysis with large particle sorter COPAS FP-1000.

### Immunofluorescence Staining

For determining pluripotency, iPS cells derived from sorted iPS cell clusters were cultured as described above. Immunofluorescence staining for the pluripotency markers TRA-1-60, TRA-1-81, OCT4 and NANOG was performed as described (Toledo et al., 2021). To assess trilineage iPS cell differentiation, embryoid bodies (EB) were generated by LHU and further cultured in EB formation medium for 5 days (Toledo et al., 2021). EB were then transferred to gelatin coated slides and cultured for 12 days in KO-DMEM supplemented with 20% FCS (PAN Biotech), 100 U/ml penicillin, 100 μg/ml streptomycin, 2 mM l-glutamine (all Thermo Fisher Scientific). Medium change was performed every second day. Immunofluorescence staining for trilineage markers α-fetoprotein (AFP), β-tubulin III (TUJ-1) and T-box transcription factor 3 (TBX3) was performed as described (Sontag et al., 2017). Antibodies used are listed in Supplementary Table S1.

### Fluorescence-Activated Cell Sorting (FACS) Analysis

Flow cytometry analysis was performed with FACS Canto II (BD Bioscience) and data analysis was done with FlowJo software (Tree Star). Antibodies used for FACS are listed in Supplementary Table S1.

### Cell Cluster Sorting of iPS Cell Colonies and EB

Analysis and sorting of cell clusters (iPS cell clusters and EB) was performed with large particle sorter COPAS FP-1000. Key parameters for optimization of cell cluster collection were “Drop Width”, “Sort Delay” and “Minimum Separation Width”. “Minimum Time of Flight (ToF)” was set as cell cluster size cut-off threshold. Cell clusters were characterized by optical density and fluorescence intensity and sorted as they exit the flow cell by an air sorting mechanism. Desired cell clusters were collected as described for each experiment.

### Automation of EB-Based Hematopoietic Differentiation

iPS cell clusters on MEF feeder were generated manually or by automation with the LHU and transferred to 6-well Clear Flat Bottom Ultra-Low Attachment plates (Corning) for EB formation. Hematopoietic differentiation was performed as previously described (Kovarova et al., 2010; Toledo et al., 2021). Briefly, EB were seeded in gelatin-coated (0.1%, Sigma Aldrich) 6-well plates (30–50 EB per well) in 2 ml of StemPro-34 SFM supplemented with 100 U/ml penicillin, 100 μg/ml streptomycin (all Thermo Fisher Scientific), 100 ng/ml stem cell factor (SCF), 50 ng/ml fms-related tyrosine kinase 3 ligand (FLT3L) and 30 ng/ml interleukin 3 (IL-3, all Peprotech), and 10 ng/ml interleukin 6/soluble interleukin 6 receptor fusion protein (hyper-IL-6, Fischer et al., 1997). Complete medium change was performed every third day. Hematopoietic cells, which appeared as suspension cells after 2–3 weeks, were harvested by gently rinsing the well with PBS. Alternatively, after 5 days in suspension culture, EB were subjected to cell cluster sorting with COPAS FP-1000. Sorted EB were then subjected to hematopoietic differentiation as above.

### Histological Analysis

iPS cell-derived hematopoietic cells were centrifuged on glass slides with Shandon Cytospin 4 cytocentrifuge (Thermo Fisher Scientific) followed by fixation with methanol at room temperature. Cells were stained with Diff Quik (Medion Diagnostics) followed by mounting with Entellan (Merck). Image acquisition and analysis was performed with Leica DMRX microscope (Leica) and Leica Application Suite software (Leica Microsystems), respectively. ImageJ was used for image handling.

### Gene Expression Analysis by RT-qPCR

RNA isolation was performed with NucleoSpin RNA Kit (Macherey-Nagel, Düren, Germany) following the manufacturer´s instructions. cDNA was synthesized using MultiScribe High Capacity cDNA Reverse Transcriptase Kit (Thermo Fisher Scientific). RT-qPCR was performed on StepOnePlus Real Time cycler with FAST SYBR Green master mix (Thermo Fisher Scientific). Sequences of primers used in the present study are listed in Supplementary Table S2.

### Generation of EB From Micro-contact Printed Vitronectin Arrays

EB generation from feeder-free iPS cell cultures was performed with micro-contact printed (µprinted) vitronectin arrays and automated by LHU. Briefly, polydimethylsiloxane stamps with circular features of 600 µm in diameter were used to pattern the surface of 6-well tissue culture plates with vitronectin arrays. iPS cells were seeded on patterned plates by LHU and cultured in StemMACS iPS-Brew XF (Miltenyi Biotech) (Elsafi Mabrouk et al., 2022). iPS cell colonies were cultured for up to 11 days and the rate of colony detachment from µprinted arrays was quantified daily.

### Calculation of Cell Confluency

Confluency of MEF feeder was calculated from phase contrast image scans of the automated EVOS Fluorescence Digital Inverted Microscope with modified PHANTAST algorithm (Jaccard et al., 2014). The algorithm was modified to cope with large images by implementing the core features in C++ using OpenCV. The images are split up in tiles, processed in parallel and then merged again. The phase contrast region in the center of wells was selected by a circular mask and areas recognized as MEF were color-coded in red. Confluency is defined as number of red pixels with respect to the number of total pixels in the circular masked area (Supplementary Figure S4C). Confluency values are provided as xml-file and the segmentation as overlay image.

### Statistical Analysis

Statistical analyses were performed in Prism 7 (GraphPad). The statistic test used, and *p*-values are indicated in the respective figure legends.

## Results

**FIGURE 1:**
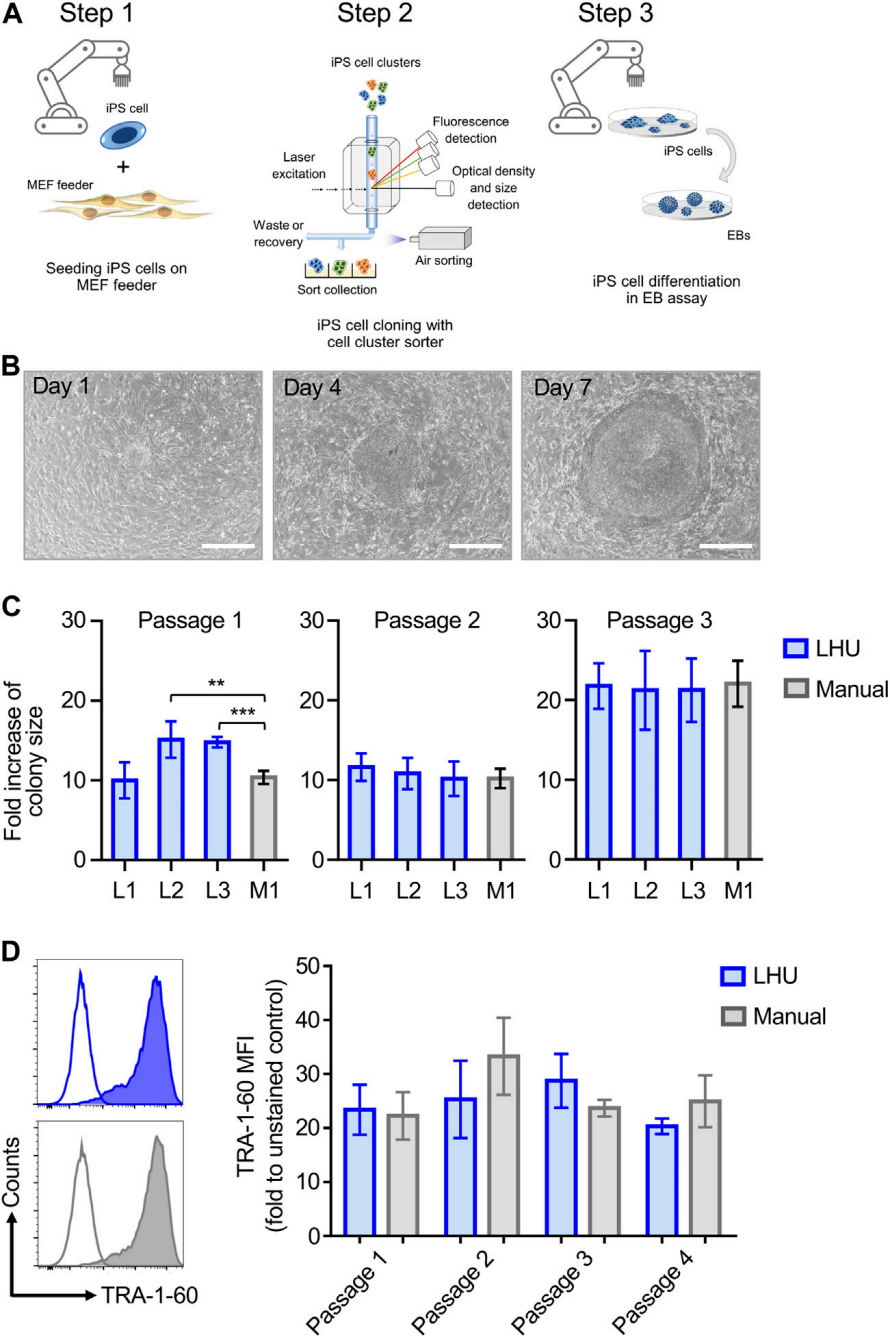
Automated iPS cell culture on MEF feeder. **(A)** Schematic representation of iPS cell culture on MEF feeder, iPS cell cloning with cell cluster sorter and iPS cell differentiation through formation of EB (Steps 1, 2 and 3, respectively). **(B)** Representative phase contrast microscopy images of the same iPS cell colony from automated culture at day 1, 4 and 7 after passaging. Scale bar: 650 µm. **(C)** The growth of iPS cell colonies cultured manually (M1, grey bars) or by the LHU (L1-L3, blue bars) was monitored for three passages and each passage was cultured for 7 days. Bars (±SD) show the averaged fold increase in colony size of four selected colonies in each experiment (*n* = 4). Colony size was determined with a custom-made python algorithm (see Supplementary Material S1 and Methods). Fold increase of colony size was calculated as the ratio of colony size on day 7 relative to day 1. **p < 0.01, ***p < 0.001. **(D)** Left: Representative histogram plots of TRA-1-60 expression on iPS cells cultured by automated handling (LHU; blue) and manual handling (Manual; gray). Empty plots show unstained cells, filled plots show stained cells. Right: TRA-1-60 mean fluorescence intensity (MFI) values obtained for iPS cells cultured by automated handing (LHU, blue, *n* = 3) and manual handling (Manual, gray, n = 3) over three consecutive passages.

The reliable and robust automation of iPS cell culture is key to high throughput application of iPS cells in disease modeling and drug screening. This involves 1) iPS cell culture, the 2) isolation of individual clonal iPS cell lines and 3) iPS cell differentiation into the desired cell types (Steps 1–3, Figure 1A).

Step 1 Automated iPS cell culture maintains iPS cell pluripotency

To this aim, we established an optimized liquid handling unit (LHU) protocol for automated culture of iPS cells on MEF feeder layer or on feeder-free vitronectin-coated plates (see Materials and Methods). We developed preprogrammed modules to automate all steps of MEF feeder layer seeding, generating confluent and homogenous MEF feeder-coated wells with high reproducibility (Supplementary Figures S1A,B; Supplementary Figures S4A,B). Homogenous MEF feeder layers were readily obtained (Supplementary Figures S4B, C) Next, we applied a similar modular preprogramming approach for culture of iPS cells on MEF feeder layer (Figures 1B–D; Supplementary Figures S2A, B; Supplementary Figures S5A–E). First, we confirmed that automation of iPS cell culture did not affect iPS cell morphology or growth, as the increase in colony size was comparable for automated and manual cultures throughout three consecutive passages (Figures 1B,C, Supplementary Figures S5A–C). Second, we demonstrated that automation of iPS cell passaging allowed to reproducibly control iPS cell cluster size by defining the number of resuspension cycles performed by the LHU (Supplementary Figures S2A; Supplementary Figures S5D, E). Third, we verified that automated cultivation maintained pluripotency of iPS cells, as similar surface expression level of the pluripotency marker TRA-1-60 was observed in automated and manual cultures (Figure 1D; Supplementary Figures S6A, B).

Step 2 iPS cell cloning is readily performed with cell cluster sorter

The establishment of iPS cell lines after reprogramming or genetic engineering (i.e. by CRISPR-Cas9 technology) relies on the isolation of individual iPS cell colonies, which develop from a single cell, thus generating a colony where all cells are clonal and share the same genetic information (Chen and Pruett-Miller, 2018; Singh, 2019). Frequently, this is accomplished by manually picking iPS cell colonies under a microscope, a time-consuming task (e.g., about 24 colonies can be manually picked in 30–45 min) that requires trained personnel and nevertheless has variable outcomes (Paull et al., 2015). Automation approaches for identification and isolation of single iPS cell colonies have been reported but require the complex integration of microscopic evaluation of iPS cell culture with an automated mechanical device that performs colony picking (Haupt et al., 2012; Elanzew et al., 2020). Here, we took advantage of the user-friendly cell cluster sorter COPAS FP-1000 for the isolation of clonal iPS cell lines in a bulk approach.

**FIGURE 2:**
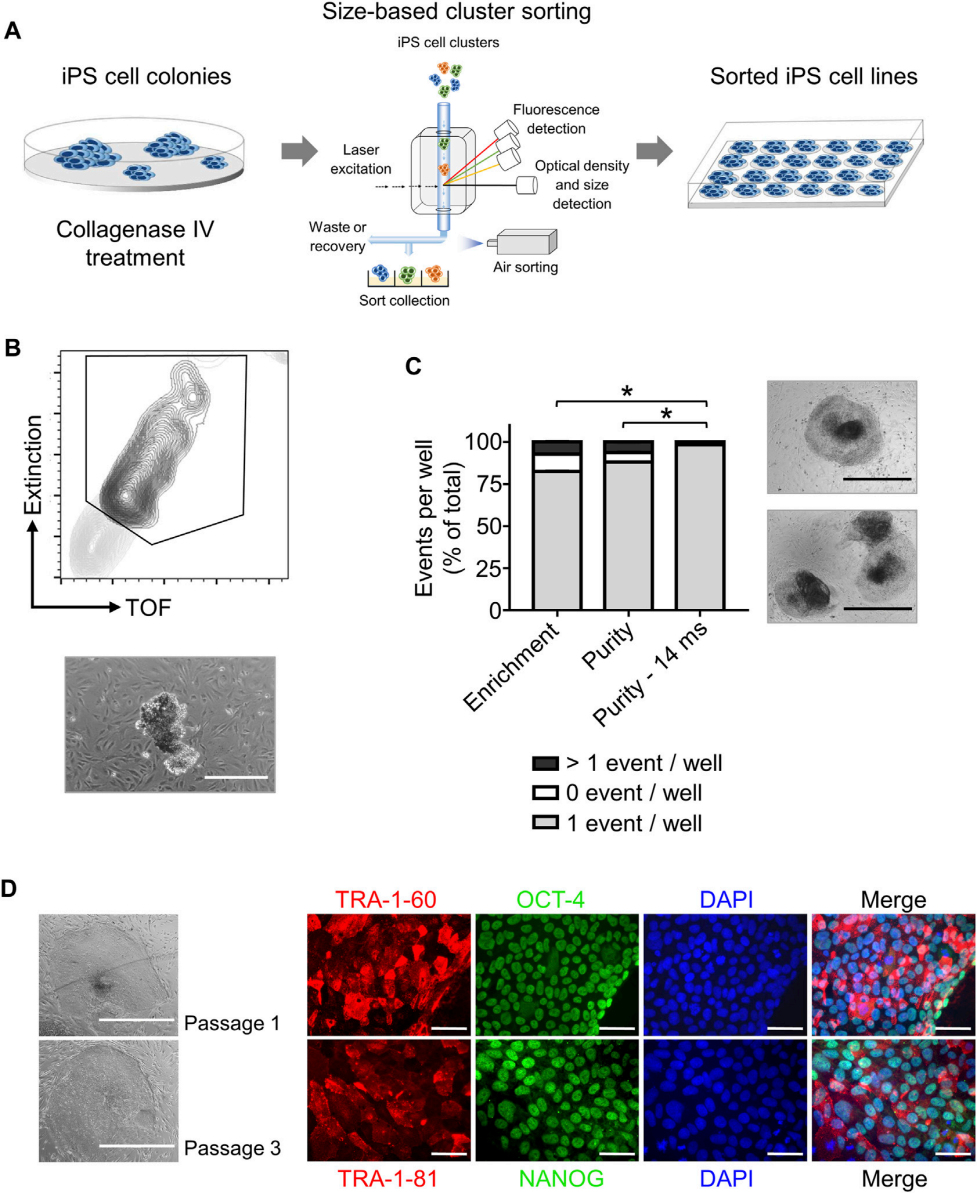
High throughput automated sorting of iPS cell clusters enables the generation of iPS cell lines without compromising pluripotency. **(A)** Schematic representation of experimental design. iPS cells are cultured on MEF feeder in 10 cm dishes until a confluency of 70% is reached. iPS cell colonies are detached by collagenase IV treatment and sorted by large particle sorter COPAS FP-1000 into a 24 well-plate. **(B)** Representative gating strategy (top) used to sort iPS cell clusters and representative image of an iPS cell cluster after sorting (bottom, scale bar: 500 µm). **(C)** Quantification of sorting efficiency for each sorting method used (enrichment, purity, or purity—14 ms) based on the number of iPS cell clusters (events) per well in a 24-well plate. Purity method with optimized delay time of 14 ms resulted in most of the wells containing only one iPS cell cluster (event) allowing clonal expansion of iPS cells. Phase contrast microscopy images on the right show iPS cell colony morphology 2 days after cluster sorting of one event per well (top) and >1 event per well (bottom). Scale bar: 1 mm. Statistical analysis was performed with Welch´s *t*-test comparing the numbers of wells containing one event after sorting with different protocols: enrichment (*n* = 3), purity (*n* = 6) or purity with delay of 14 ms (*n* = 8). * = p< 0.0001. **(D)** Representative phase contrast microscopy images of iPS cells generated after sorting showing morphology of pluripotent cells after one and three passages (Left, scale bar: 1 mm). Pluripotency of sorted iPS cells was further confirmed by immunofluorescence staining for the pluripotency surface markers TRA-1-60 and TRA-1-81 and the pluripotency associated transcription factors OCT4 and NANOG. Nuclei are stained with DAPI. *n* = 1. Scale bar: 100 µm.

iPS cells were seeded as single cells on MEF feeder at low cell density and expanded to colonies, and iPS cell colonies were harvested by collagenase IV treatment. iPS cell clusters were obtained by gentle mechanical agitation and individual iPS cell clusters were sorted into 24 well plates, precoated with MEF feeder layer, based on particle time of flight (TOF) and extinction coefficient (Figures 2A, B). The sorting efficiency of iPS cell clusters per well was determined by visual inspection and microscopy (Figure 2B). We tested different sorting protocols (enrichment or purity) and optimized sorting parameters (sort delay in purity vs. purity-14 ms), and determined settings that allowed us to achieve over 98.4 ± 2% of wells containing an individual iPS cell cluster (Figure 2C). Sorted iPS cell clusters successfully attached on MEF feeder and generated iPS cell colonies (Figure 2C). Importantly, with our automated approach 100 iPS cell lines were readily obtained within 10 min, which outperforms the time-consuming manual iPS cell colony picking. Additionally, the iPS cell colonies obtained by cell cluster sorter maintained pluripotency, as assessed by morphology and immunofluorescence staining for the pluripotency markers TRA-1-60, TRA-1-81, OCT4 and NANOG (Figure 2D).

Next, we proceeded to demonstrate that iPS cell clonality is preserved during sorting. To this end, iPS cell clusters were labeled with PKH26 (red) or CFSE (green), mixed and then subjected to cell cluster sorting. Individual iPS cell clusters bearing either PKH26 (red) or CFSE (green) color code, but not both, were obtained and sorted (Supplementary Figure S7A, B). Thus, our protocol can also be applied to sort iPS cell clusters labeled with different fluorochromes, a valuable tool to readily isolate genetically engineered iPS cell lines harboring reporter genes such as eGFP or tdTomato.

**FIGURE 3:**
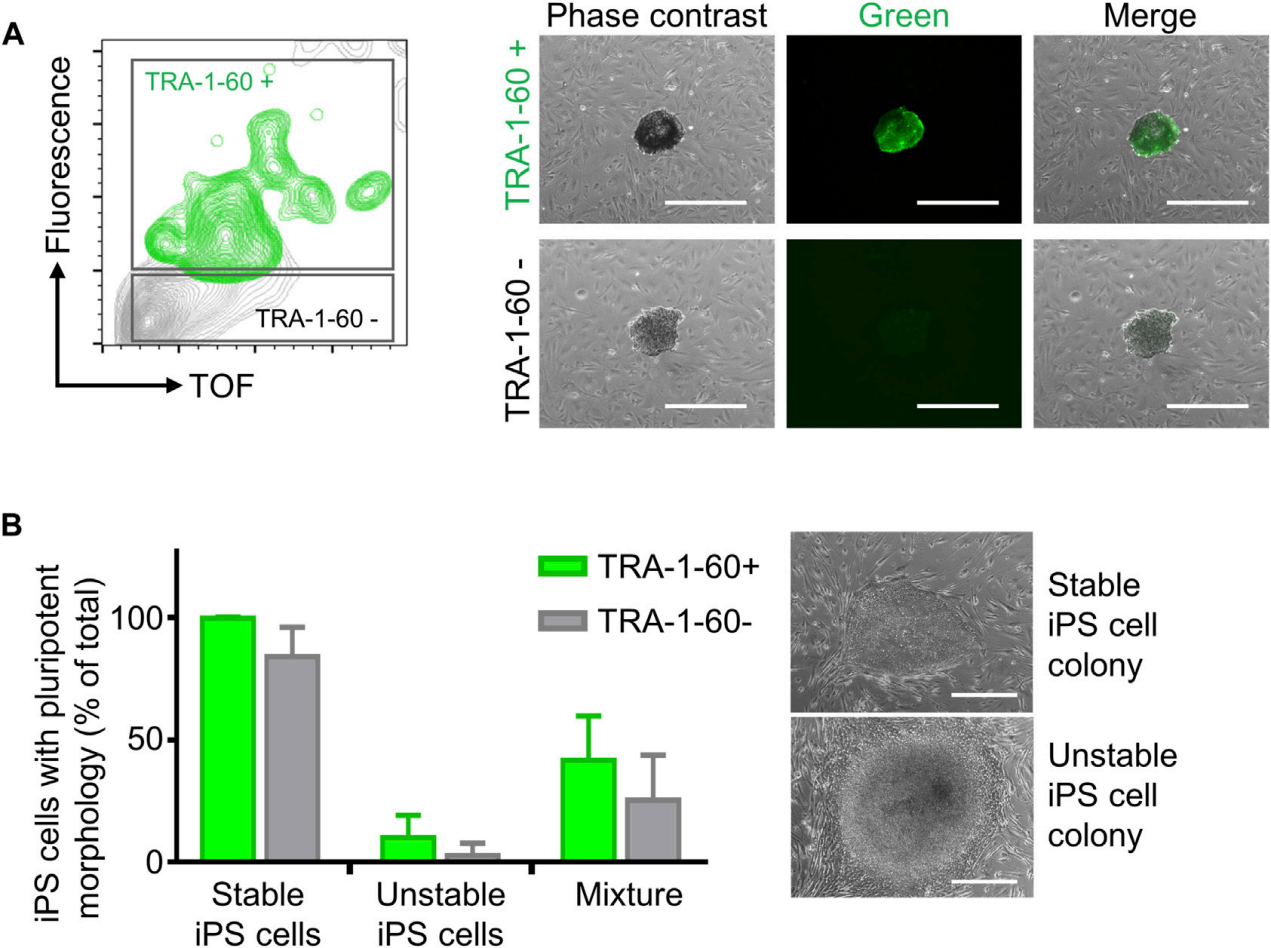
Enrichment of TRA-1-60 positive iPS cell clusters. **(A)** Representative gating strategy for sorting iPS cell clusters stained with TRA-1-60 (left). TRA-1-60 positive sorted iPS cell clusters showed homogeneous TRA-1-60 staining by fluorescence microscopy (right). Scale bar: 500 µm. **(B)** Quality assessment of TRA-1-60 positive and negative iPS cell clusters derived from stable and unstable iPS cell lines after cell cluster sorting. Mixture: Stable and unstable iPS cell cluster were mixed in 1:1 ratio, stained with TRA-1-60 and subjected to cell cluster sorting based on TRA-1-60 expression. Higher numbers of iPS cell colonies with iPS/ES cell morphology are observed in TRA-1-60 positive vs. TRA-1-60 negative populations, although not statistically significant (stable, *n* = 3, *p* = 0.0874, unstable, *n* = 3, *p* = 0.3017, mixture, *n* = 3, *p* = 0.3360). Stable and unstable cell lines were defined by their morphology and level of spontaneous differentiation in culture. Representative images of iPS cell colonies with pluripotent and unstable/differentiated morphology are shown (top and bottom images respectively, scale bar: 500 µm).

Reprogramming of somatic cells into iPS cells is a complex process and frequently incompletely reprogrammed iPS cells are obtained. Such incompletely reprogrammed iPS cells are unstable and exhibit reduced staining for pluripotency markers (Cahan and Daley, 2013; Pomeroy et al., 2016). Thus, we combined cell cluster sorting of iPS cells with TRA-1-60 live-cell staining to enrich for iPS cells with high expression of this pluripotency marker. TRA-1-60-positive iPS cell clusters were efficiently separated from TRA-1-60-negative or -low clusters and enrichment of TRA-1-60-positive iPS cell clusters was confirmed by fluorescence microscopy (Figure 3A). We evaluated this approach with a stable iPS cell line (good iPS cell morphology and high TRA-1-60 surface expression) and an unstable iPS cell line (differentiated morphology of iPS cell colonies and variable TRA-1-60 surface expression). After cell cluster sorting, higher numbers of iPS cell colonies with pluripotent morphology were observed in the TRA-1-60-positive fraction and as expected, higher numbers of iPS cell colonies with pluripotent morphology were obtained for the stable iPS cell line (Figure 3B).

Step 3 Automated differentiation of iPS cells into hematopoietic cells

iPS cells are valuable tools for disease modeling and drug discovery since they represent an essentially unlimited cell source and are able to differentiate into virtually any cell type of the human body (Rowe and Daley, 2019). Frequently, a key step in iPS cell differentiation towards the desired cell type is the formation of embryoid bodies (EB), an aggregate of pluripotent stem cells kept in suspension culture, where the initial steps of lineage commitment and differentiation occur. Following EB formation, further differentiation of cells towards a specific tissue and/or cell type is induced by a defined cocktail of cytokines and/or differentiation factors (Guo et al., 2020).

**FIGURE 4:**
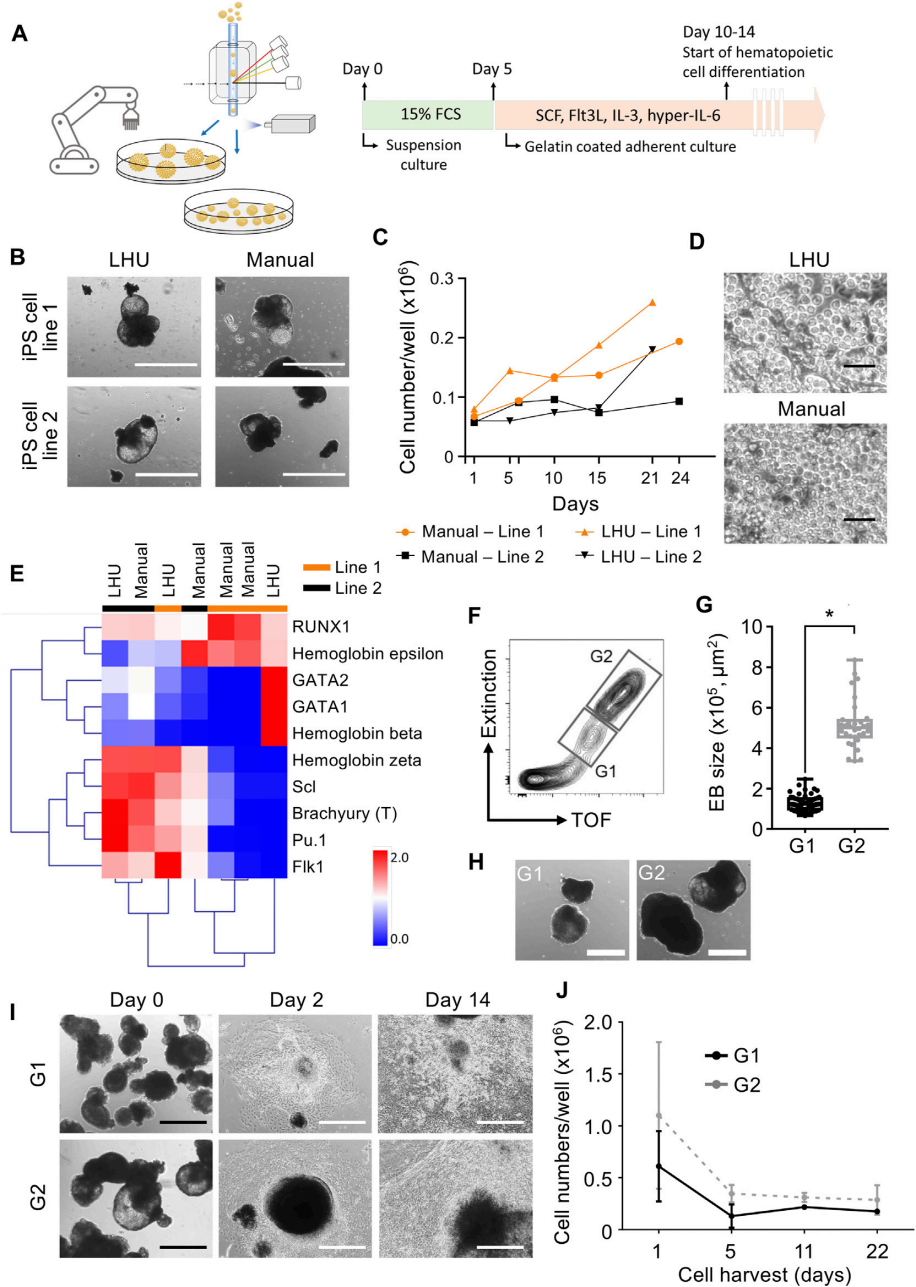
Automated generation and sorting of iPS cell-derived embryonic bodies (EB) for hematopoietic differentiation. **(A)** Schematic representation of automated iPS cell hematopoietic differentiation pipeline. In the automated platform, iPS cells are expanded and EB are generated. Size based selection of EB is performed with the cell cluster sorter. EB formation is performed with an initial suspension culture step in 15% FCS supplemented mesoderm inducing medium, followed by an adherent culture step in hematopoietic supporting medium supplemented with SCF, Flt3L, IL-3 and hyper-IL-6. **(B)** Representative images of EB generated manually or by automation with the LHU. Scale bar: 1,000 µm. **(C,D)** Production of hematopoietic cells from EB generated manually (*n* = 2) or by automation (*n* = 2) with the LHU for two iPS cell lines (line 1 and line 2) **(C)**. Representative phase contrast microscopy images of suspension hematopoietic cells produced by EB generated manually or by automation with the LHU **(D)**. Scale bar: 50 µm. **(E)** RT-qPCR gene expression profile of hematopoietic cells derived from manually (*n* = 4) or by automation (n = 3) generated EB, produced from two iPS cell lines (line 1 and line 2) as in **(C)**. Gene expression values were subjected to bidirectional hierarchical clustering and are shown in heatmap format (red and blue, high and low gene expression, respectively). Hematopoietic cells differentiated from the adherent cell layer after 3–6 days of culture were used. Expression of the early mesodermal transcription factor Brachyury (T) and the early hematopoietic cytokine receptor Flk1, and a panel of key hematopoietic transcription factors (RUNX1, GATA1, GATA2, Scl and Pu.1) and the hemoglobins beta, epsilon and zeta are shown. **(F–H)** Sorting of EB based on EB size (TOF, time of flight). Representative gating strategy for sorting EB using gate 1 (G1: EB with area range of 0.65–2.46 × 10^5^ μm^2^) and gate 2 (G2: EB with area range of 3.36–8.35 × 10^5^ μm^2^) **(F)**. Quantification of sorted EB based on size in gates G1 and G2 (n = 4 for both, *: *p* = 0.0001) **(G)**. EB size was calculated with custom-made python algorithm (Supplementary Material S1 and Methods). Representative images of EB sorted in G1 and G2 **(H)**. Scale bar: 500 µm. **(I, J)** G1 and G2 sorted EB were subjected to hematopoietic differentiation on gelatin coated culture plates. Representative images of sorted G1 and G2 EB seeded on gelatin-coated plates for hematopoietic differentiation right after sorting (day 0), after 2 and 14 days of culture **(I)**. Scale bar: 500 µm. Quantification of hematopoietic cells produced from EB sorted in G1 (dark gray, *n* = 3) and G2 (light gray, *n* = 3) **(J)**.

Therefore, we applied the same modular preprogramming approach with the LHU for automation of EB formation (Figure 4A). No morphological differences were observed between EB produced by automation or manually from two iPS cell lines (Figure 4B). EB generated by automation differentiated towards the three germ layers showing that their differentiation potential was fully maintained (Supplementary Figures S8, S9). EB were then further differentiated towards the hematopoietic lineage and no differences in endothelial/stromal tissue development or hematopoietic cell production were observed for manually or automatically processed EB (Figures 4C, D; Supplementary Figure S8). Gene expression profiling of the hematopoietic cells produced confirmed their hematopoietic identity. Importantly, unsupervised clustering indicated that hematopoietic cells cluster by iPS cell line of origin rather than by the method of cultivation (manually vs. LHU, Figure 4E).

EB size impacts on iPS cell differentiation potential and efficiency (Messana et al., 2008; Hong et al., 2010; Zeevaert et al., 2020). Thus, we investigated whether EB size has an impact in hematopoietic differentiation of iPS cells in our automated platform. EB were subjected to cell cluster sorting based on TOF and extinction coefficient using a 2-gate strategy. Phase contrast microscopy evaluation confirmed efficient size-based separation of EB with size ranging from 0.65–2.46 × 10^5^ μm^2^ in gate 1 (G1) or 3.36–8.35 × 10^5^ μm^2^ in gate 2 (G2) (Figures 4F–H). Particles of size below 0.65 × 10^5^ μm^2^ were excluded. Further hematopoietic differentiation of G1 and G2 sorted EB showed efficient endothelial/stromal tissue development and hematopoietic cell production (Figures 4I, J; Supplementary Figures S10A,B).

Additionally, a novel and innovative approach to control EB size is to use micro-contact printing (µCP) (Elsafi Mabrouk et al., 2022). iPS cell colony size and hence EB size are controlled by the size of the vitronectin area printed on the tissue culture plate. To this end, we adapted µCP EB formation to our automated platform. Automated culture of iPS cells on µCP vitronectin arrays yielded iPS cell colonies confined to the vitronectin-coated area of 600 µm in diameter (Supplementary Figures S11A). During culture iPS cell colonies started to detach in EB-like structures after 7 days, with full detachment occurring after 11 days (Supplementary Figures S11A–C).

In summary, we show that our automated platform stands as an efficient and robust system for iPS cell culture and differentiation. Cell cluster sorting allowed for analysis and sorting of iPS cell clusters and establishment of clonal iPS cell lines from bulk populations. In addition, EB formation is efficiently automated in our platform and combined with cell cluster sorting to enrich for EB with specific size. The EB generated by automation were successfully differentiated towards hematopoietic cells with efficiency and quality indistinguishable from manual processing. Finally, our automated platform output has high upscaling potential, as seven 6-well plates can be handled simultaneously by the LHU and several rounds of automated iPS cell culture and cell cluster sorting can be performed daily (Supplementary Table S3).

## Discussion

Automation of iPS cell generation, culture, genetic manipulation, and differentiation into cells/tissues of interest is key to the large scale application of patient and disease specific iPS cells in disease modeling and drug screening (Daniszewski et al., 2018; De Masi et al., 2020). Human iPS cells enable the recapitulation of patient and disease heterogeneity, which in turn requires a large number of iPS cell lines to faithfully model a particular pathology (Ben Jehuda et al., 2018; McTague et al., 2021; Toledo et al., 2021). In addition, the CRISPR/Cas9 technology stands as a precise, particularly easy and versatile genetic engineering tool for iPS cells (Hotta and Yamanaka, 2015), which requires the respective machinery and workflow for downstream processing. All this asks for the further development of automated platforms for iPS cell culture and differentiation.

Here, we report on a user-friendly, modular preprogrammed, automated platform for iPS cell culture on a feeder-free or MEF based culture system. We developed a library of preprogrammed automation steps that can be easily selected and combined by the user to create customized protocols. This approach allowed us to use the same platform also for blood cell differentiation. Importantly, our modular system requires only basic training to operate the software and almost no programming skills from the users. Furthermore, our pipeline relies on two main operational blocks: the liquid handling unit (LHU) and the cell cluster sorter, thereby reducing establishment and operational costs. The flexibility of our platform did not compromise its robustness and sterility, as no contamination occurred throughout the entire study.

Several studies reported on the automation of iPS cell culture, addressing automation of iPS cell reprogramming, clonal isolation, induced clonal stability, and differentiation towards several tissues (Haupt et al., 2012; Konagaya et al., 2015; Paull et al., 2015; Coston et al., 2020; Elanzew et al., 2020; Tristan et al., 2020). Our work contributes to these efforts by providing a novel approach for the generation and differentiation of iPS cell lines. By combining our automated iPS cell cultivation platform with a cell cluster sorting we demonstrate that: 1) clonal iPS cell lines are readily generated by iPS cell cluster sorting with high efficiency and without compromising pluripotency, a key requirement for the generation of a large number of reprogrammed iPS cell lines or genetically CRISPR/Cas9-engineered iPS cell lines, 2) fluorescently labeled iPS cells are efficiently sorted as iPS cell clusters without compromising cell viability and pluripotency and 3) iPS cell-derived EB are efficiently sorted by size without compromising EB viability and differentiation potential. Of note, iPS cell-derived EB are efficiently differentiated towards the hematopoietic lineage providing material for downstream studies. We also envision iPS-cell derived organoids to be generated and selected by our pipeline, a topic that will be addressed in future studies.

Another attractive approach is the combination of our automated pipeline for iPS cell culture and EB generation with bioreactor-based hematopoietic differentiation strategies, which have shown high yields of functional hematopoietic cells with therapeutic value (Ackermann et al., 2018). In addition, our pipeline can also be used to speed-up the clonal selection of genetically modified iPS cell lines that are further expanded in high density bioprocessors for downstream applications (Manstein et al., 2021).

Our study paves the way for the future development of specific automation protocols aiming the large-scale establishment and CRISPR/Cas9-mediated genetic engineering of iPS cell lines. This includes iPS cell differentiation towards desired cells and tissues, which are most suitable for disease modeling and drug screening studies.

## Data Availability

The raw data supporting the conclusion of this article will be made available by the authors, without undue reservation.
