# Supplementary material for: Cell Cluster Sorting in Automated Differentiation of Patient-specific Induced Pluripotent Stem Cells Towards Blood Cells
Source: Front Bioeng Biotechnol. 2022 May 12;10:755983. doi: 10.3389/fbioe.2022.755983 (PMC9157239; doi:10.3389/fbioe.2022.755983)
Supplement: Supplementary file 3 [file DataSheet1.pdf]

A

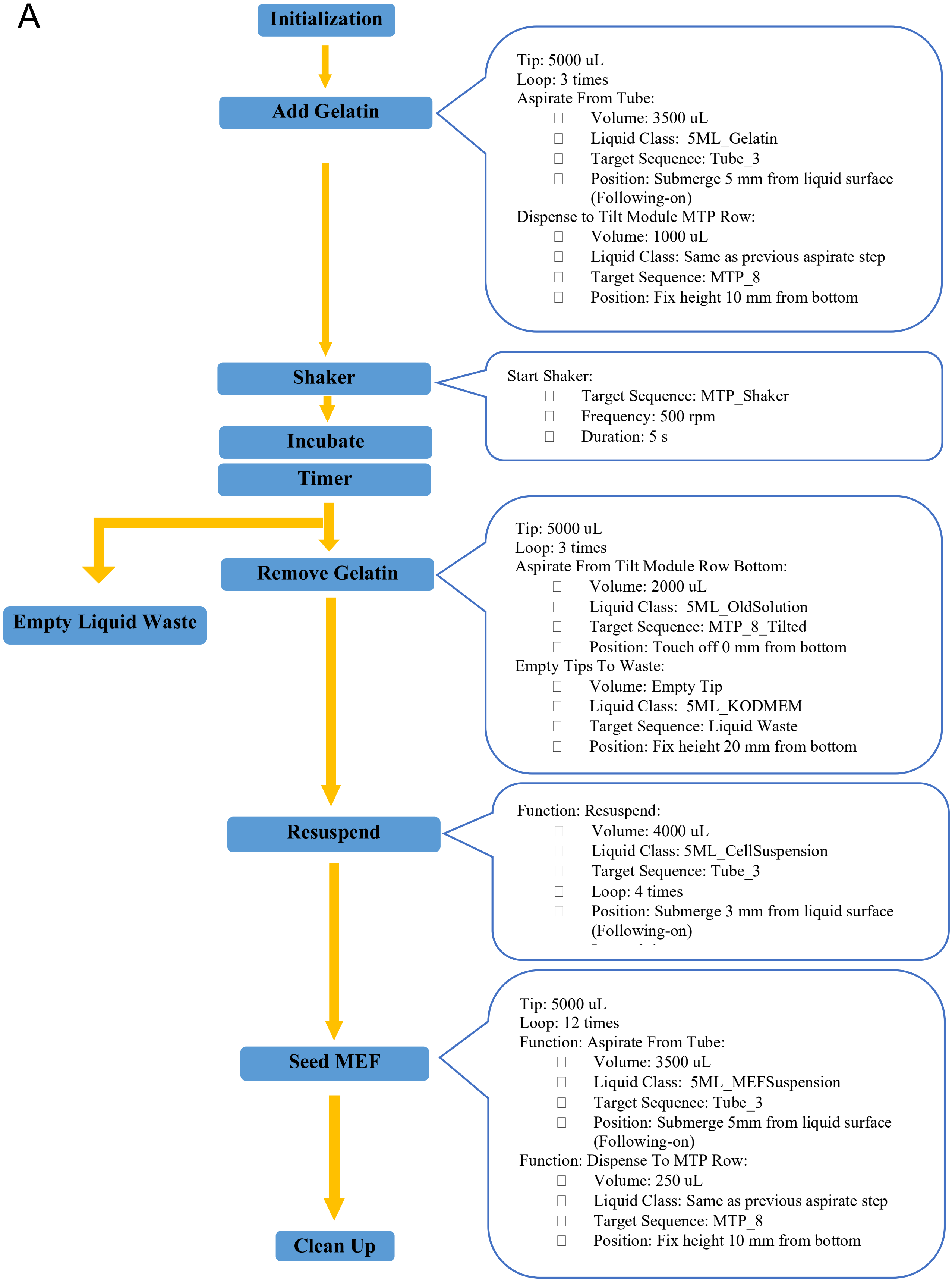
Initialization
Add Gelatin
Tip: 5000 uL
Loop: 3 times
Aspirate From Tube:
Volume: 3500 uL
Liquid Class: 5ML_Gelatin
Target Sequence: Tube_3
Position: Submerge 5 mm from liquid surface (Following-on)
Dispense to Tilt Module MTP Row:
Volume: 1000 uL
Liquid Class: Same as previous aspirate step
Target Sequence: MTP_8
Position: Fix height 10 mm from bottom
Shaker
Start Shaker:
Target Sequence: MTP_Shaker
Frequency: 500 rpm
Duration: 5 s
Incubate
Timer
Remove Gelatin
Empty Liquid Waste
Tip: 5000 uL
Loop: 3 times
Aspirate From Tilt Module Row Bottom:
Volume: 2000 uL
Liquid Class: 5ML_OldSolution
Target Sequence: MTP_8_Tilted
Position: Touch off 0 mm from bottom
Empty Tips To Waste:
Volume: Empty Tip
Liquid Class: 5ML_KODMEM
Target Sequence: Liquid Waste
Position: Fix height 20 mm from bottom
Resuspend
Function: Resuspend:
Volume: 4000 uL
Liquid Class: 5ML_CellSuspension
Target Sequence: Tube_3
Loop: 4 times
Position: Submerge 3 mm from liquid surface (Following-on)
Seed MEF
Tip: 5000 uL
Loop: 12 times
Function: Aspirate From Tube:
Volume: 3500 uL
Liquid Class: 5ML_MEFSuspension
Target Sequence: Tube_3
Position: Submerge 5mm from liquid surface (Following-on)
Function: Dispense To MTP Row:
Volume: 250 uL
Liquid Class: Same as previous aspirate step
Target Sequence: MTP_8
Position: Fix height 10 mm from bottom
Clean Up

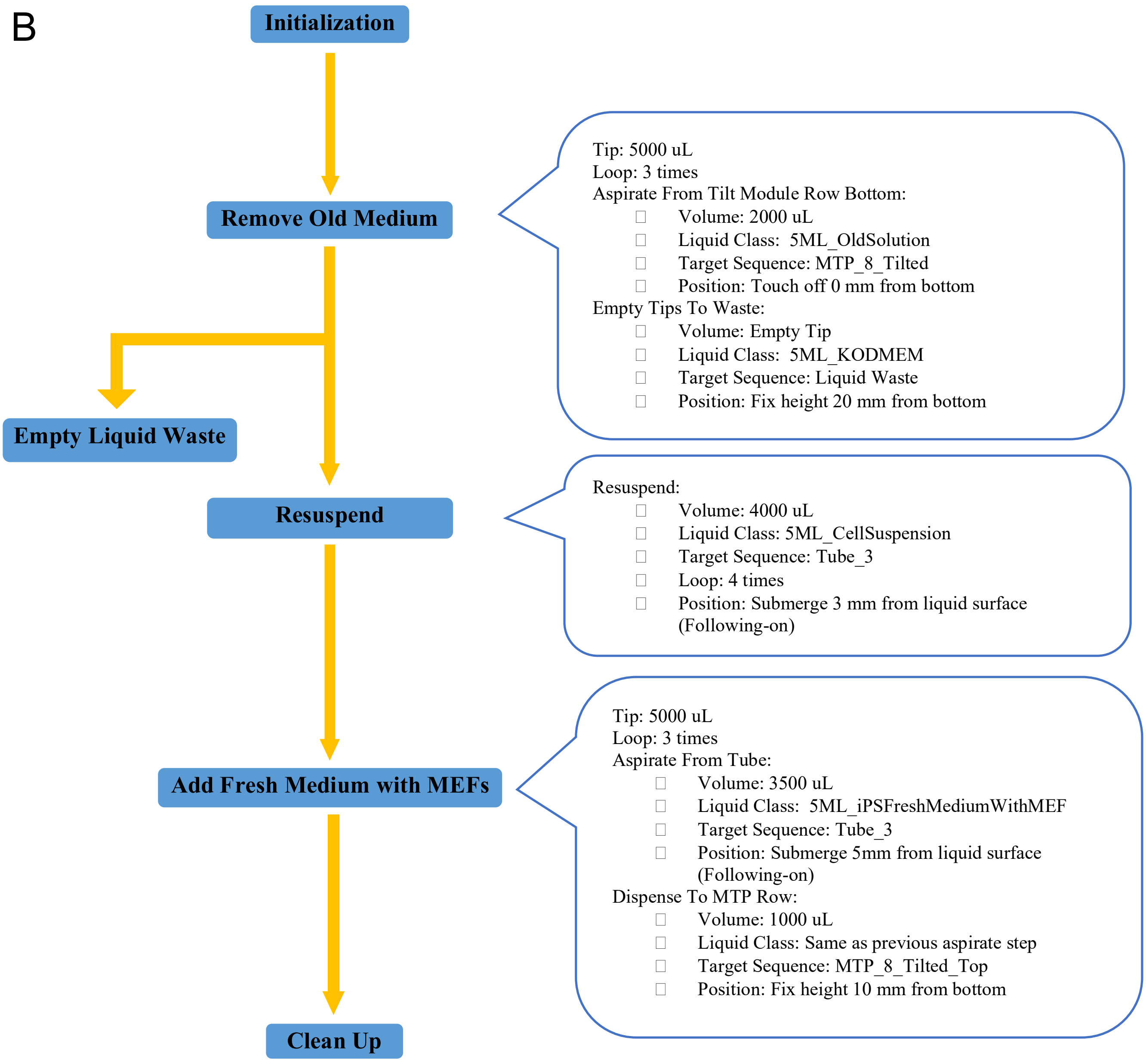

Supplementary Figure S1. LHU MEF culture operating system.

(A) “hiPS_MEF_Coating” flowchart with detailed parameters. The flowchart represents the sequence of operations to prepare MEF feeder plates for iPS cell culture. First, a gelatin layer was deposited on culture plate by LHU and second MEF were seeded on gelatin layer.

(B) “hiPS_MEF_Reseed” flowchart with detailed parameters. The flowchart represents the sequence of operations to reseed MEF on iPS cell cultures with MEF feeder to maintain a functional feeder layer during culture. Old culture medium was replaced by MEF suspension.

A

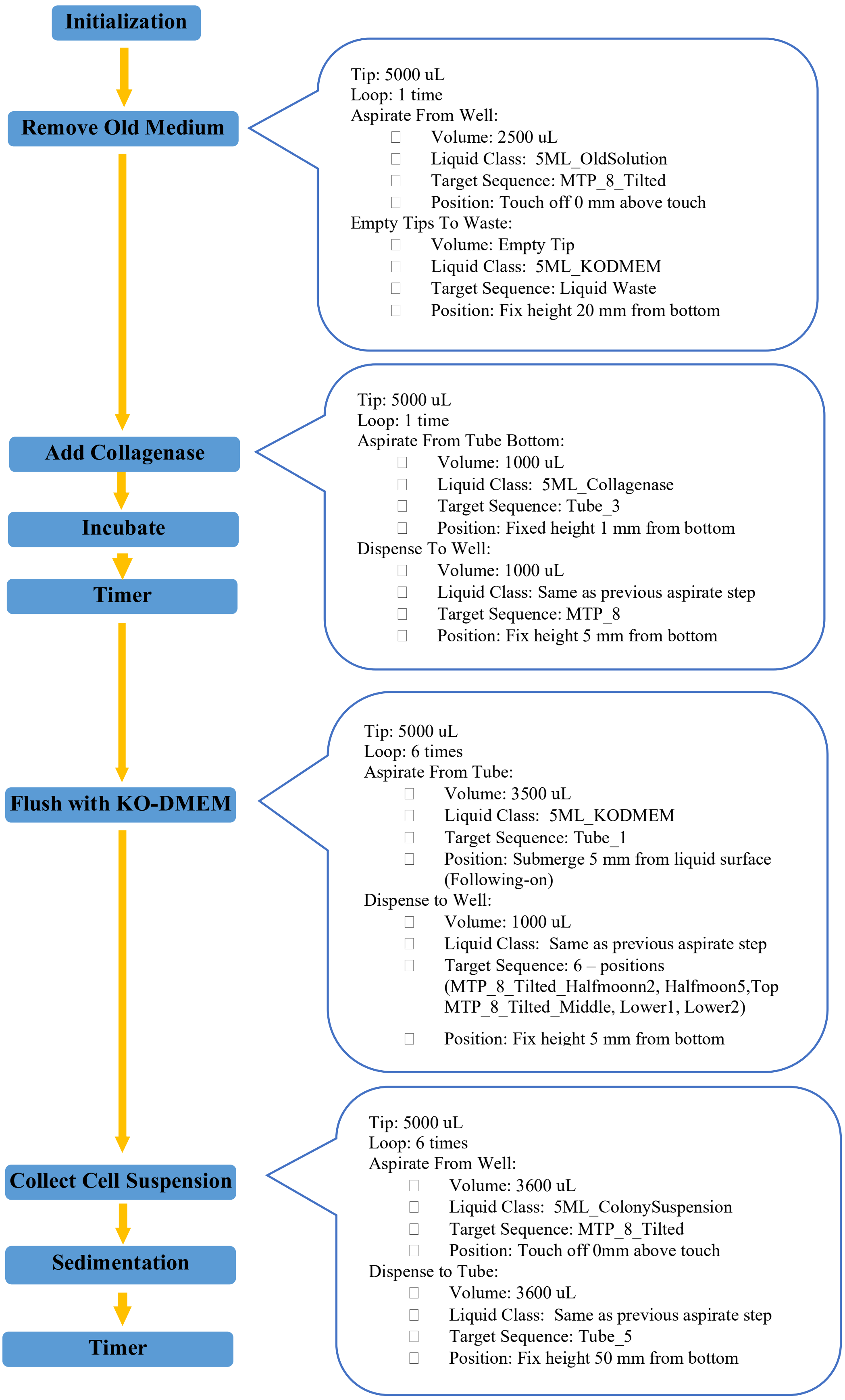

Initialization
Remove Old Medium
Tip: 5000 uL
Loop: 1 time
Aspirate From Well:
Volume: 2500 uL
Liquid Class: 5ML_OldSolution
Target Sequence: MTP_8_Tilted
Position: Touch off 0 mm above touch
Empty Tips To Waste:
Volume: Empty Tip
Liquid Class: 5ML_KODMEM
Target Sequence: Liquid Waste
Position: Fix height 20 mm from bottom
Add Collagenase
Tip: 5000 uL
Loop: 1 time
Aspirate From Tube Bottom:
Volume: 1000 uL
Liquid Class: 5ML_Collagenase
Target Sequence: Tube_3
Position: Fixed height 1 mm from bottom
Dispense To Well:
Volume: 1000 uL
Liquid Class: Same as previous aspirate step
Target Sequence: MTP_8
Position: Fix height 5 mm from bottom
Incubate
Timer
Flush with KO-DMEM
Tip: 5000 uL
Loop: 6 times
Aspirate From Tube:
Volume: 3500 uL
Liquid Class: 5ML_KODMEM
Target Sequence: Tube_1
Position: Submerge 5 mm from liquid surface (Following-on)
Dispense to Well:
Volume: 1000 uL
Liquid Class: Same as previous aspirate step
Target Sequence: 6 – positions (MTP_8_Tilted_Halfmoonn2, Halfmoon5,Top MTP_8_Tilted_Middle, Lower1, Lower2)
Position: Fix height 5 mm from bottom
Collect Cell Suspension
Tip: 5000 uL
Loop: 6 times
Aspirate From Well:
Volume: 3600 uL
Liquid Class: 5ML_ColonySuspension
Target Sequence: MTP_8_Tilted
Position: Touch off 0mm above touch
Dispense to Tube:
Volume: 3600 uL
Liquid Class: Same as previous aspirate step
Target Sequence: Tube_5
Position: Fix height 50 mm from bottom
Sedimentation
Timer

A (continued)

Remove Supernatant

Tip: 5000 uL
Loop: 1 time
Aspirate From Tube Bottom:
- Volume: 4500 uL
- Liquid Class: 5ML_Supernatant
- Target Sequence: Tube_5
- Position: Fixed height 5mm from bottom

Empty Tips To Waste:
- Volume: Empty Tip
- Liquid Class: 5ML_KODMEM
- Target Sequence: Liquid Waste
- Position: Fix height 20 mm from bottom

Break Up Colonies

Tip: 5000 uL
Loop: 2 times
Aspirate From Tube:
- Volume: 3500 uL
- Liquid Class: 5ML_iPSFreshMedium
- Target Sequence: Tube_7
- Position: Submerge 5mm from liquid surface (Following-on)

Dispense to Tube:
- Volume: 3500 uL
- Liquid Class: Same as previous aspirate step
- Target Sequence: Tube_5
- Position: Fix height 50mm from bottom

Resuspend:
- Volume: 4000 uL
- Liquid Class: 5ML_ColonySuspension2
- Target Sequence: Tube_5
- Loop: 6 times
- Position: Submerge 3 mm from liquid surface (Following-on)
- [illegible]

Distribute Colonies

Tip: 5000 uL
Loop: 12 times
Resuspend:
- Volume: 4000 uL
- Liquid Class: 5ML_ColonySuspension2
- Target Sequence: Tube_5
- Position: Submerge 3 mm from liquid surface (Following-on)
- Loop: 2 times

Aspirate From Tube Bottom:
- Volume: 3500 uL
- Liquid Class: 5ML_ColonySuspension1
- Target Sequence: Tube_5
- Position: Fixed height 1 mm from bottom

Dispense To MTP Row:
- Volume: 250 uL
- Liquid Class: Same as previous aspirate step
- Target Sequence: MTP_3
- Position: Fix height 10 mm from bottom

Clean Up

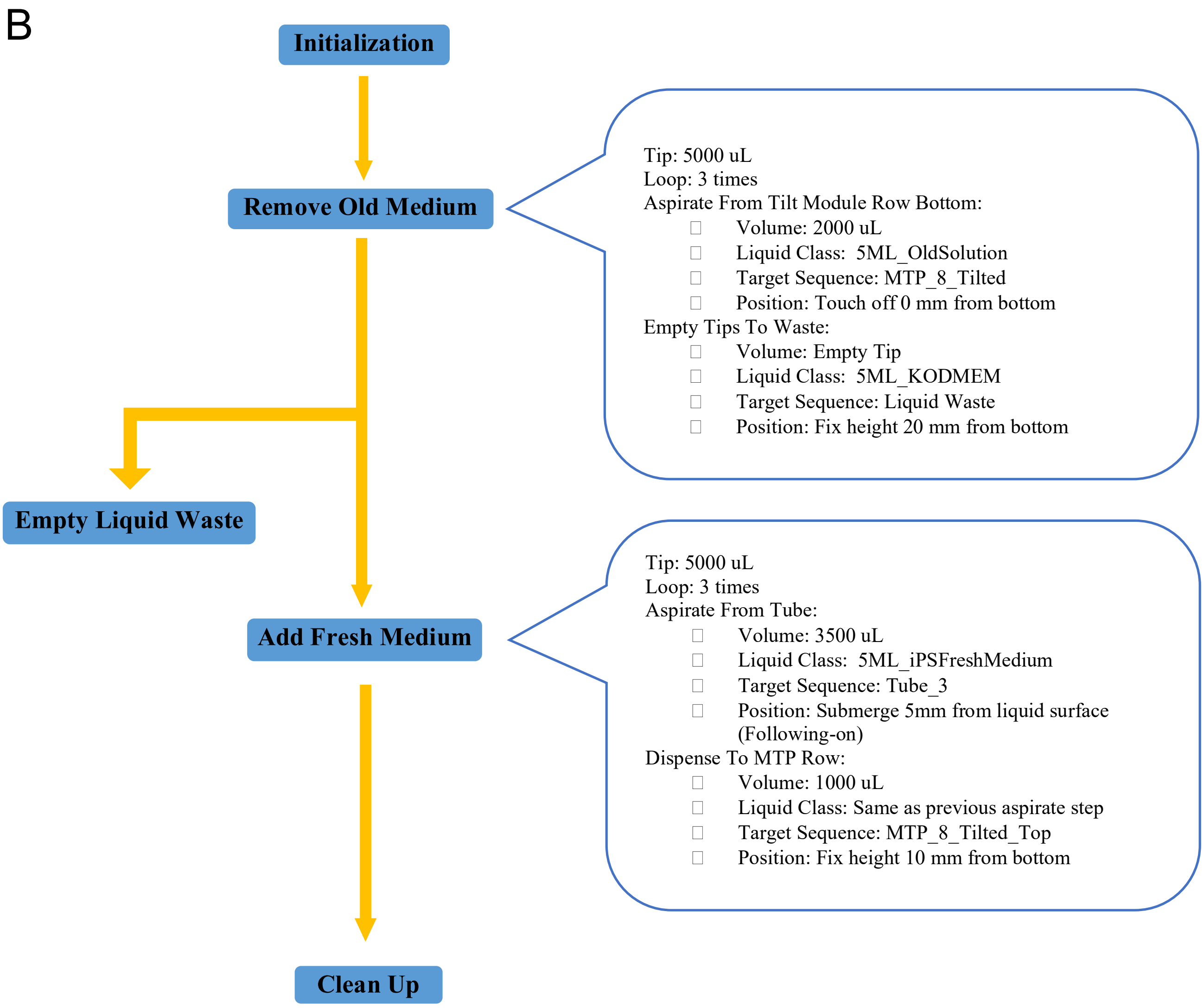

Supplementary Figure S2. LHU iPS cell culture operating system.

(A) “hiPS_Passaging” flowchart with detailed parameters. The flowchart represents the sequence of operations for passaging iPS cells on MEF feeder. Collagenase IV was added to iPS cell culture plate and incubated at 37°C and 5% CO_2 to detach iPS cell colonies from MEF feeder layer. Detached colonies in KO-DMEM were collected and broken into appropriate sizes by resuspension. The resulting colonies were evenly distributed on new MEF feeder for the next culture passage.

(B) “hiPS_Medium_Change” flowchart with detailed parameters. The flowchart represents the sequence of operations to change medium for iPS cell culture. Old medium was removed from culture plate and fresh medium was added.

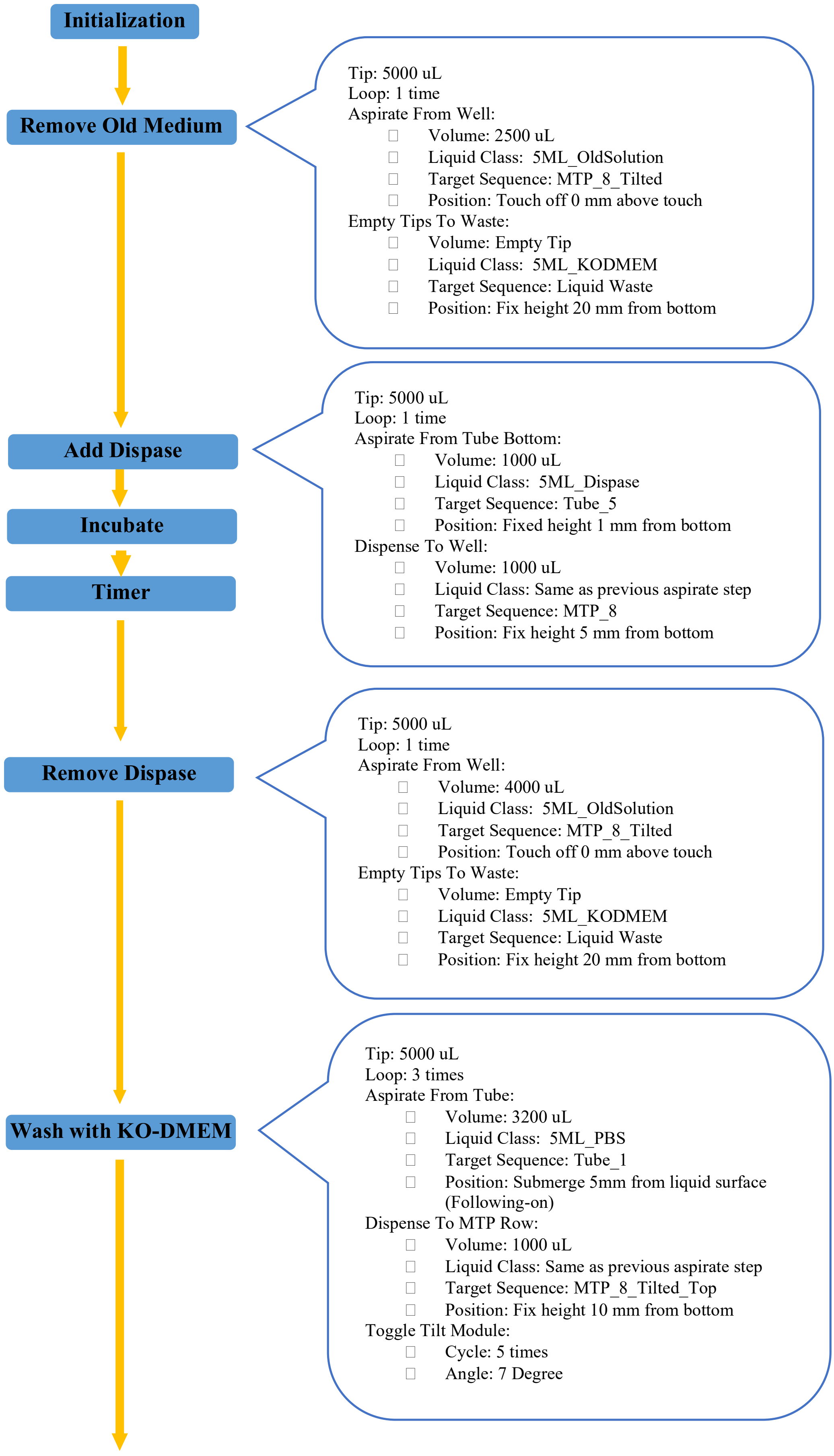
Initialization
Remove Old Medium
Tip: 5000 uL
Loop: 1 time
Aspirate From Well:
Volume: 2500 uL
Liquid Class: 5ML_OldSolution
Target Sequence: MTP_8_Tilted
Position: Touch off 0 mm above touch
Empty Tips To Waste:
Volume: Empty Tip
Liquid Class: 5ML_KODMEM
Target Sequence: Liquid Waste
Position: Fix height 20 mm from bottom
Add Dispase
Incubate
Timer
Tip: 5000 uL
Loop: 1 time
Aspirate From Tube Bottom:
Volume: 1000 uL
Liquid Class: 5ML_Dispase
Target Sequence: Tube_5
Position: Fixed height 1 mm from bottom
Dispense To Well:
Volume: 1000 uL
Liquid Class: Same as previous aspirate step
Target Sequence: MTP_8
Position: Fix height 5 mm from bottom
Remove Dispase
Tip: 5000 uL
Loop: 1 time
Aspirate From Well:
Volume: 4000 uL
Liquid Class: 5ML_OldSolution
Target Sequence: MTP_8_Tilted
Position: Touch off 0 mm above touch
Empty Tips To Waste:
Volume: Empty Tip
Liquid Class: 5ML_KODMEM
Target Sequence: Liquid Waste
Position: Fix height 20 mm from bottom
Wash with KO-DMEM
Tip: 5000 uL
Loop: 3 times
Aspirate From Tube:
Volume: 3200 uL
Liquid Class: 5ML_PBS
Target Sequence: Tube_1
Position: Submerge 5mm from liquid surface (Following-on)
Dispense To MTP Row:
Volume: 1000 uL
Liquid Class: Same as previous aspirate step
Target Sequence: MTP_8_Tilted_Top
Position: Fix height 10 mm from bottom
Toggle Tilt Module:
Cycle: 5 times
Angle: 7 Degree

Remove KO-DMEM

Tip: 5000 uL
Loop: 1 time
Aspirate From Well:

- Volume: 4000 uL
- Liquid Class: 5ML_OldSolution
- Target Sequence: MTP_8_Tilted
- Position: Touch off 0 mm above touch

Empty Tips To Waste:

- Volume: Empty Tip
- Liquid Class: 5ML_KODMEM
- Target Sequence: Liquid Waste
- Position: Fix height 20 mm from bottom

Wash with KO-DMEM

Tip: 5000 uL
Loop: 3 times
Aspirate From Tube:

- Volume: 3200 uL
- Liquid Class: 5ML_PBS
- Target Sequence: Tube_1
- Position: Submerge 5mm from liquid surface (Following-on)

Dispense To MTP Row:

- Volume: 1000 uL
- Liquid Class: Same as previous aspirate step
- Target Sequence: MTP_8_Tilted_Top
- Position: Fix height 10 mm from bottom

Toggle Tilt Module:

- Cycle: 5 times
- Angle: 7 Degree

Remove KO-DMEM

Tip: 5000 uL
Loop: 1 time
Aspirate From Well:

- Volume: 4000 uL
- Liquid Class: 5ML_OldSolution
- Target Sequence: MTP_8_Tilted
- Position: Touch off 0 mm above touch

Empty Tips To Waste:

- Volume: Empty Tip
- Liquid Class: 5ML_KODMEM
- Target Sequence: Liquid Waste
- Position: Fix height 20 mm from bottom

Add KO-DMEM

Tip: 5000 uL
Loop: 3 times
Aspirate From Tube:

- Volume: 3200 uL
- Liquid Class: 5ML_PBS
- Target Sequence: Tube_1
- Position: Submerge 5mm from liquid surface (Following-on)

Dispense To MTP Row:

- Volume: 1000 uL
- Liquid Class: Same as previous aspirate step
- Target Sequence: MTP_8_Tilted_Top
- Position: Fix height 10 mm from bottom

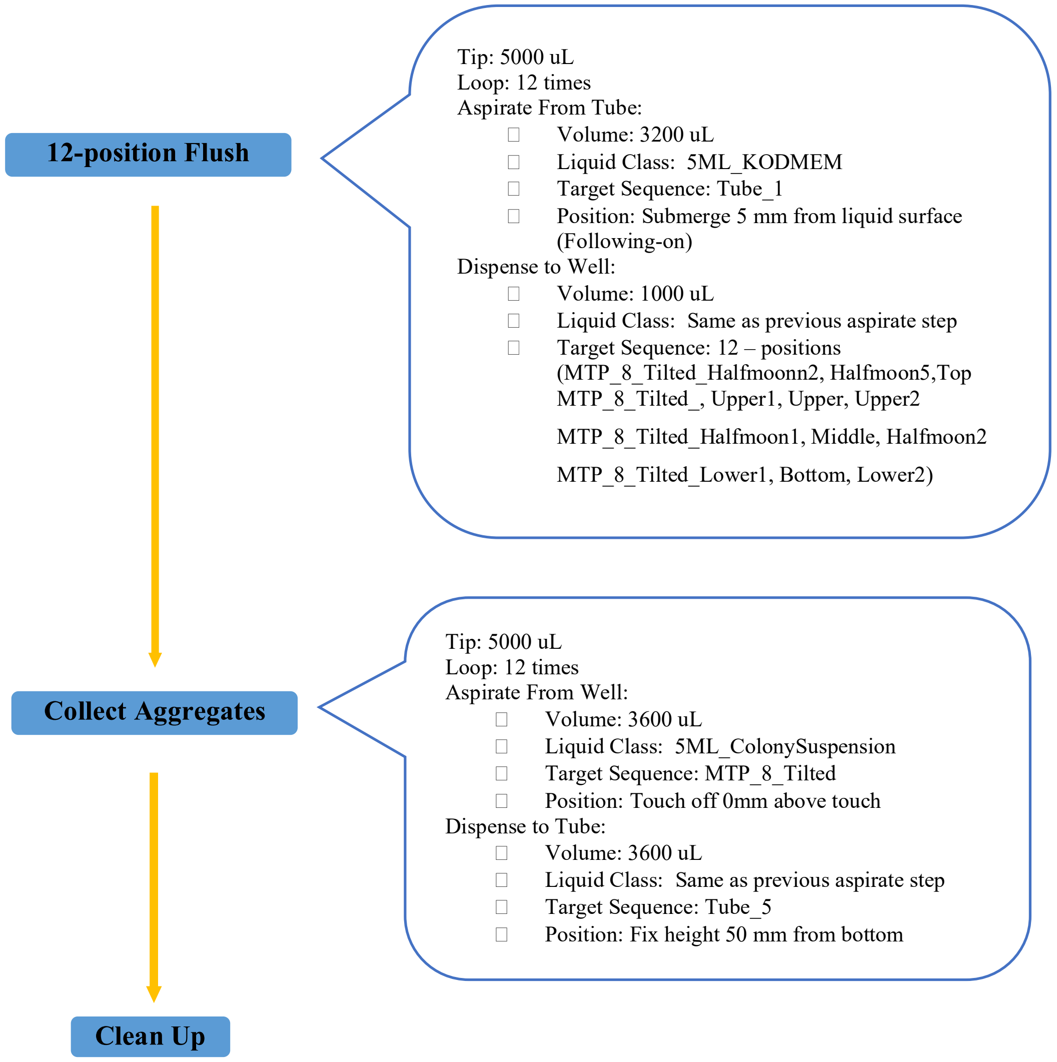

Supplementary Figure S3. LHU EB formation operating system

“EB_Formation” flowchart with detailed parameters. The flowchart represents the sequence of operations to form embryoid bodies (EB) from iPS cell colonies. iPS cell colonies were first partially detached from MEF feeder layer with dispase treatment. 12-position flush function was used to harvest iPS cell colonies. Cell clusters were suspended in medium and subjected to EB formation.

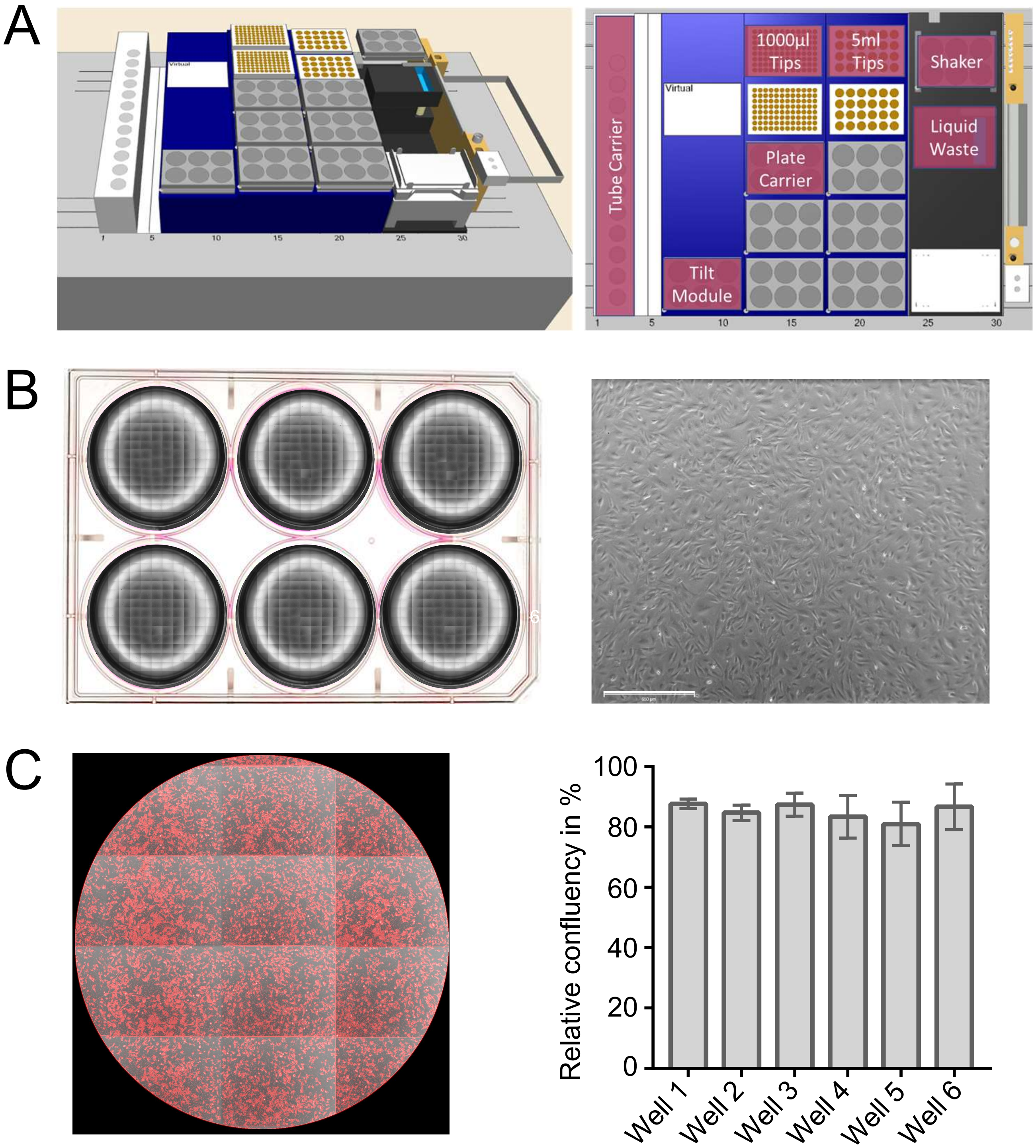

Supplementary Figure S4. Automated seeding and culture of MEF feeder layer for iPS cell culture.

(A) 3D illustration of the deck layout of the Hamilton Liquid Handling Unit (LHU) system showing all carriers and labware used in the system (left). Top view of the system deck showing major functional carriers (right): tube carrier, tilt module, plate carriers, tip carriers, shaker, liquid waste container and multiple 6-well plates (grey).

(B) MEF feeder layer in 6-well plate obtained by automated MEF seeding with LHU. MEF feeder layer in 6-well plate was scanned by EVOS FL Auto 2 microscope and individual images were assembled per well by image stitching using EVOS's built-in program (left). Representative individual EVOS phase contrast microscopy image of MEF feeder layer seeded by LHU (right). Scale bar: 650 µm.

(C) Evaluation of MEF feeder layer confluency by automated seeding. Modified "Phantast" algorithm was used for calculation of MEF feeder layer confluency (left). The well center was selected by a circular mask and areas recognized as MEF were color-coded in red. Confluency is defined as number of red pixels with respect to the number of total pixels in the circular masked area. Quantification of MEF feeder layer confluency seeded automatically (right). MEF confluency in all 6 wells of 6-well plate was routinely over 80 precent and no significant difference was observed among wells.

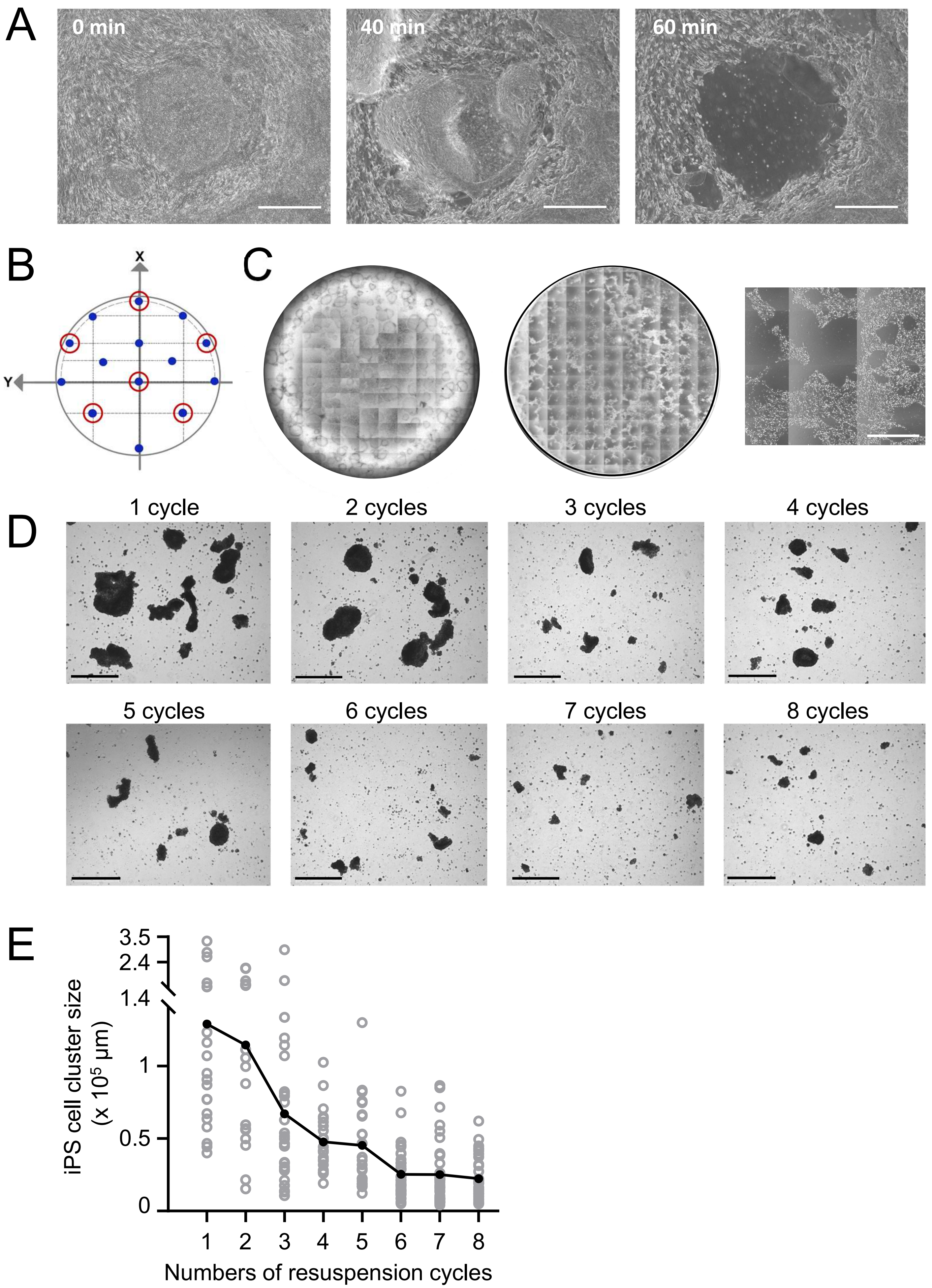

Supplementary Figure S5. Automated passaging of iPS cells on MEF feeder.

(A) Microscopy images showing iPS cell colony detachment by collagenase IV treatment over time. Sixty minutes collagenase IV treatment led to complete colony detachment and was used as standard treatment time for automated handling. Scale bar: 650 µm.

(B) LHU pipetting positions in one well of 6-well plate. Fourteen distinct positions were created and 6 positions, marked in red, were selected for iPS cell passaging.

(C) Evaluation of LHU iPS cell colony harvest after collagenase IV treatment. Scan of one well before automated passaging (left). Scan of the well after automated iPS cell colony harvest (middle). Stitched microscopy image at the center of the well after automated handling showing efficient detachment and harvest of iPS cells (right). Scale bar: 2 mm.

(D) Representative microscopy images of iPS cell cluster morphology and size after different numbers of automated resuspension cycles. Scale bar: 650 µm.

(E) Quantification of iPS cell cluster size after different numbers of automated resuspension cycles. iPS cell clusters (n=25) from each resuspension cycle group (1 to 8 cycles) were randomly chosen and their sizes were calculated. As expected, the average iPS cell cluster size and the variance among iPS cell clusters decreased with increasing cycle number.

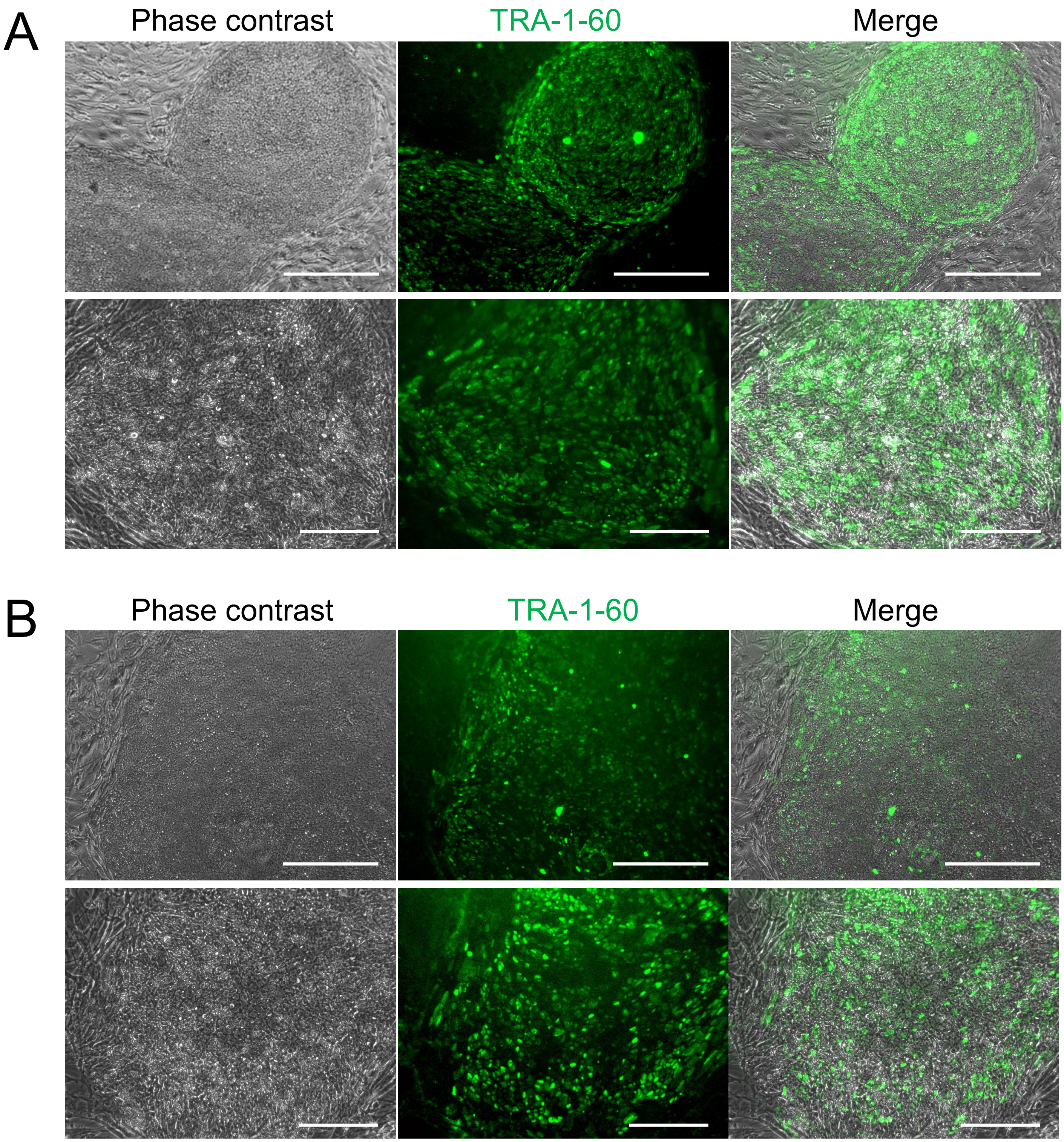

Supplementary Figure S6. Assessment of pluripotency of LHU cultured iPS cells by TRA-1-60 live-cell staining.

Representative phase contrast and fluorescence microscopy images of iPS cell colonies cultured in 6-well format by automation with the LHU (A) or manually (B). iPS cell colonies were stained for TRA-1-60 marker (green) and one representative well from LHU and one from manually handled 6-well plate are shown. Scale bar: 500 µm (top images) and 200 µm (bottom images) of panels (A) and (B).

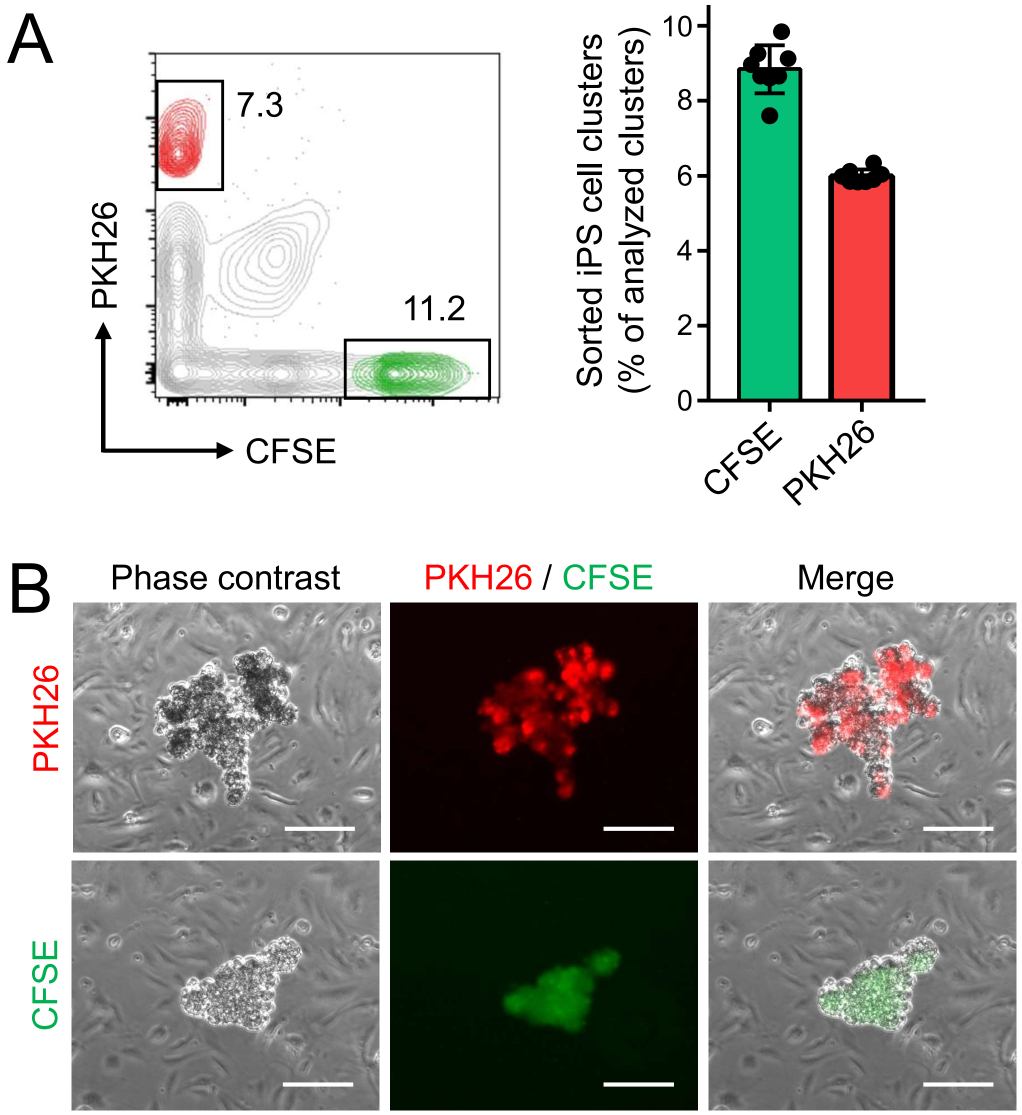

Supplementary Figure S7. Cell cluster sorting preserves clonality of fluorescently labeled iPS cell clusters.

iPS cell clusters were separately labeled with the fluorescent dyes CSFE (green) or PKH26 (red), respectively, mixed 1:1 and subjected to cell cluster sorting.

(A) Cluster sorting efficiently separates CSFE or PKH26 labeled iPS cell clusters and thus preserves clonality of clusters (left). Quantification of sorted CSFE or PKH26 labelled iPS cell clusters (right, n=8).

(B) Sorted CSFE or PKH26 labelled iPS cell clusters were seeded on MEF feeder and analyzed by phase contrast and fluorescence microscopy. Scale bar: 200 µm.

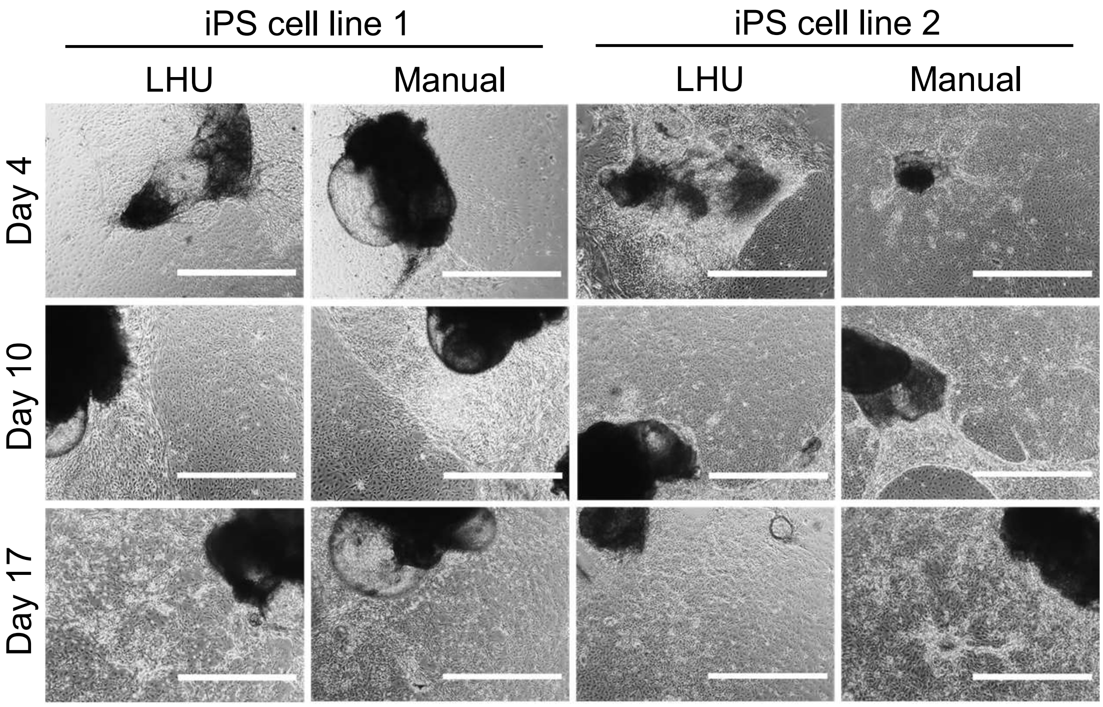

Supplementary Figure S8. EB generated by automation efficiently differentiate towards the hematopoietic lineage.

Representative images of EB produced manually or by the LHU from 2 iPS cell lines (line 1 and line 2) on days 4, 10 and 17. EB are seeded on gelatin-coated culture plates in hematopoietic differentiation medium. Endothelial and stromal tissue were observed from day 4 onwards. Suspension hematopoietic cells and clusters of hematopoietic differentiation were observed at around day 17. Scale bar: 1000 µm.

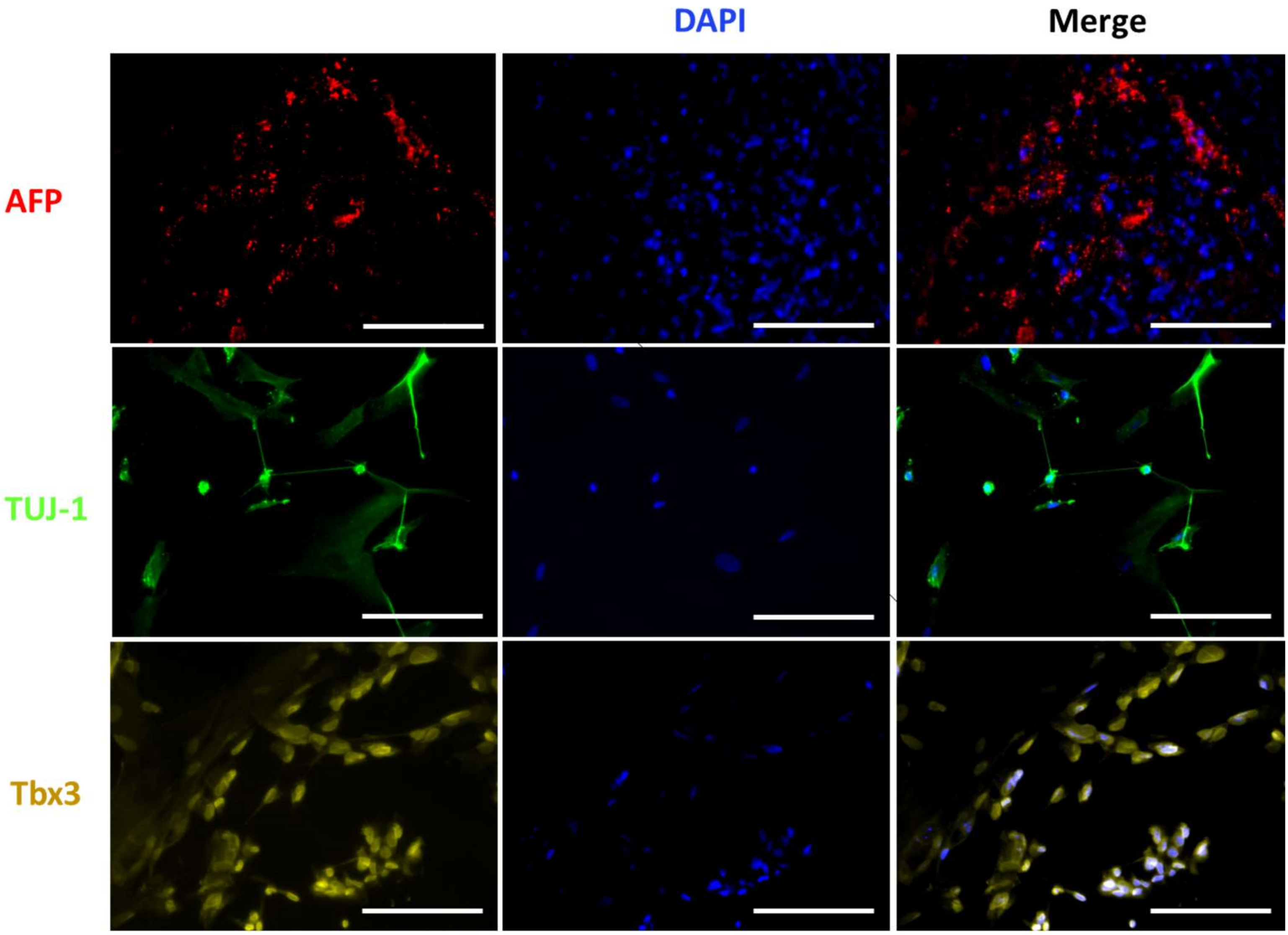

Supplementary Figure S9. EB generated by automation show trilineage differentiation capacity.

Differentiated EB were stained on day 7 after seeding on gelatin-coated plates for lineage-specific markers. AFP: alpha feta protein (endoderm). TUJ-1 (ectoderm), TBX3 (mesoderm). Nuclei were stained with DAPI. Scale bar: 100 µm.

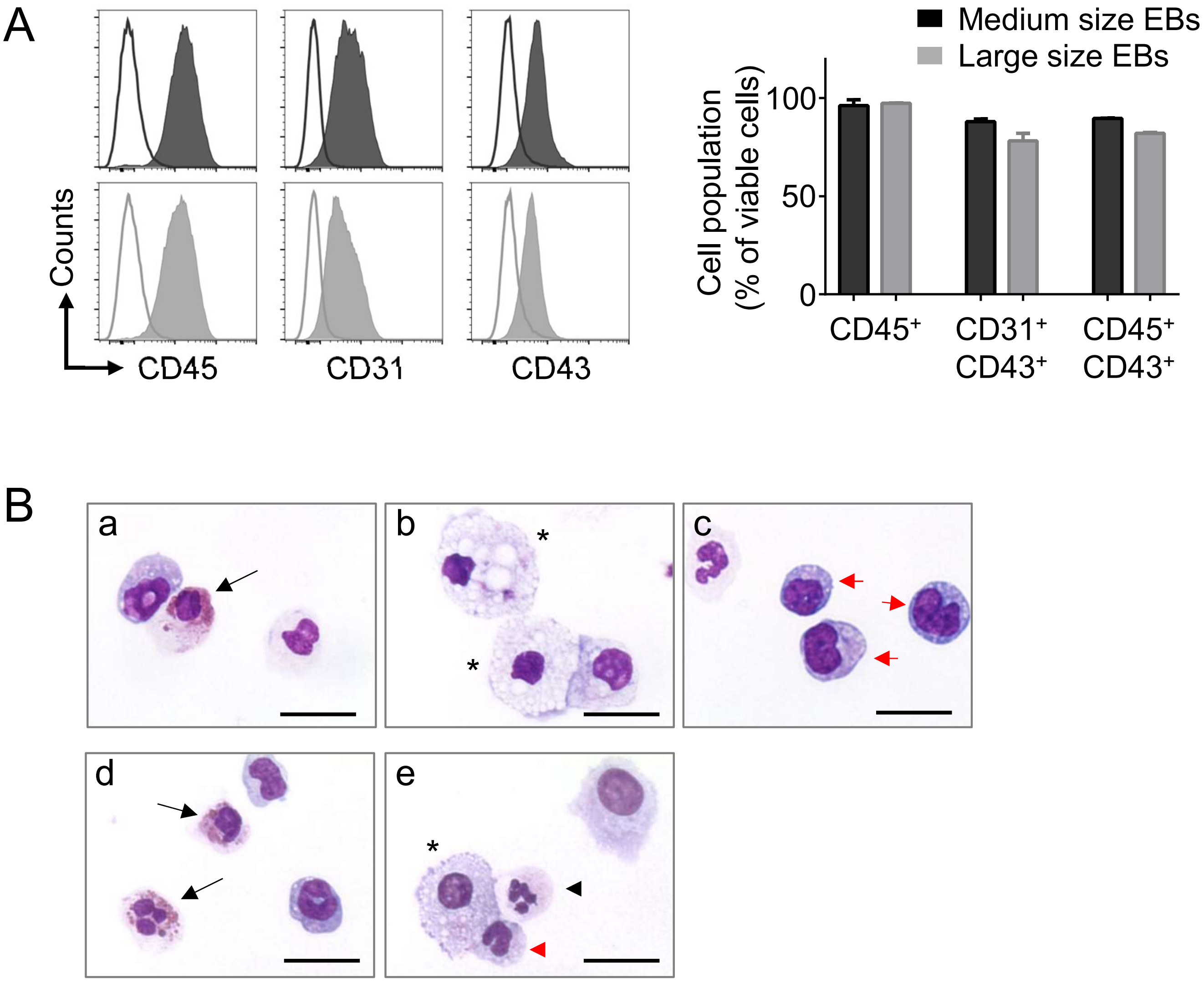

Supplementary Figure S10. Characterization of EB-derived hematopoietic cells by flow cytometry.

Medium size (dark gray) and large size EB (light gray) obtained by cell cluster sorting were differentiated into hematopoietic cells and analyzed by flow cytometry and histological staining.

(A) Filled histogram plots show cells stained for hematopoietic markers CD45, CD31 or CD43 (left). Empty histogram plots show unstained cells. Quantification of the CD45+, CD31+ CD43+ and CD45+ CD43+ hematopoietic populations (n=2) (right).

(B) Representative cytospin images of EB-derived hematopoietic cells (line 2). Cells with granulocytic (a, d, black arrow), macrophage (b, e, asterisk) and myeloid progenitor (c, red arrow), monocyte (e, red arrowhead), and neutrophil (e, black arrowhead) morphology were observed. Scale bar: 25 μm.

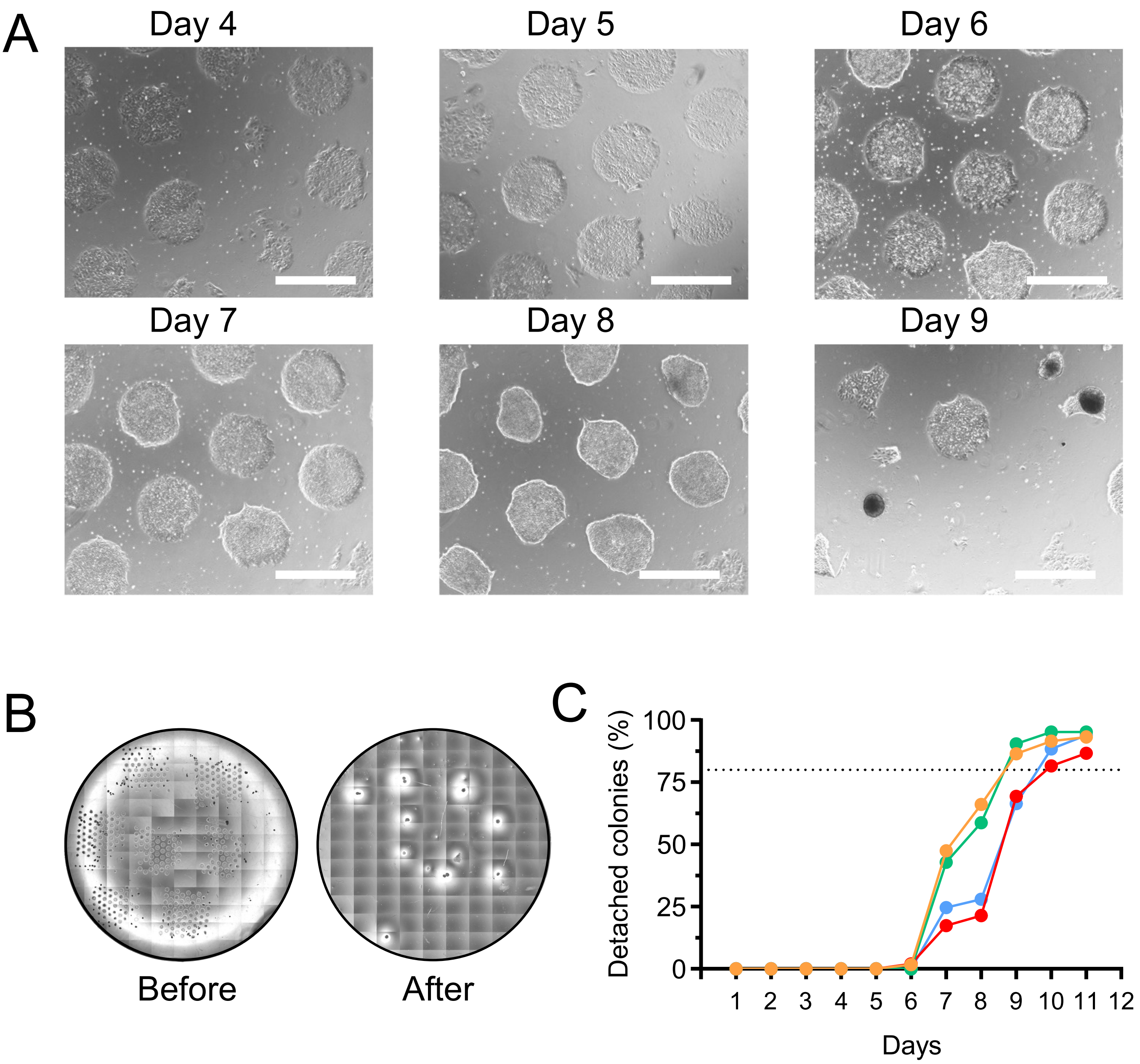

Supplementary Figure S11. Automated generation of EB from vitronectin µprinted arrays.

(A) Representative phase contrast microscopy images of iPS cell colonies cultured on vitronectin-coated µprinting plates showing changes in morphology from day 4 after seeding to day 9. Spontaneously detached iPS cell colonies at day 9 are shown. Scale bar: 650 µm.

(B) Evaluation of efficiency of LHU iPS cell colony collection for µprinting iPS cell culture. Left: Scan of well before automated colony collection. Right: Scan of well after automated iPS cell colony collection.

(C) Quantification of spontaneous iPS cell colony detachment rates cultured on µprinting plates. Most colonies detached within 3 days after initial detachment from day 7-9 onwards

“SizeCal” Python (V3.6.7) code

```
%matplotlib inline
import cv2
import matplotlib.image as mpimg
import numpy as np
from matplotlib import pyplot as plt
# imports libraries
 img = cv2.imread(' ',0)
 plt.rcParams["figure.figsize"] = (20,15)
 # imports source image
 ret2,th2 = cv2.threshold(img,35,255,cv2.THRESH_BINARY)
 temp,contours,hierarchy = cv2.findContours(th2.copy(), cv2.RETR_TREE, cv2.CHAIN_APPROX_NONE)
 # finds the edge of iPS cell colonies
for i in range(len(contours)):
    cnt = contours[i]
    line = cv2.drawContours (line,[cnt],0, (255,0,255), 31)
    print(cv2.contourArea(cnt))
# draws lines around detected iPS colonies and prints iPS cell colonies’ area
plt.subplot(1, 2, 1)
plt.imshow(img, cmap = 'gray')      # prints source image
plt.subplot(1, 2, 2)
plt.imshow(line, cmap = 'gray')      # prints detected iPS colonies
```

Supplementary Material S1. Custom-made Python algorithm to determine colony size (please see Figure 1C).
